# Multi-Stimuli-Responsive Hydrogels: From Strategic Design to Biomedical Applications

**DOI:** 10.3390/gels12100906

**Published:** 2026-10-08

**Authors:** Sijing Yi, Hao-Ying Li, Ru Chang

**Affiliations:** 1 Department of Basic Science, Shanxi Agricultural University, Taigu 030801, China; yisijing@sxau.edu.cn; 2 Institute of Pharmaceutical Science, King’s College London, Franklin Wilkins Building, 150 Stamford Street, London SE1 9NH, UK; 3 School of Automation and Software Engineering, Shanxi University, Taiyuan 030031, China

**Keywords:** multi-stimuli-responsiveness, smart hydrogels, biomaterials, controlled drug delivery, wound healing, tissue engineering

## Abstract

Multi-stimuli-responsive hydrogels have emerged as versatile biomaterial platforms for biomedical applications owing to their capacity to recognize and respond to multiple environmental signals. Such integration can provide responsiveness to signals that vary spatially and temporally. However, the effectiveness of such integrated systems requires more than simply incorporating signal-recognition components: the response characteristics of individual components must be preserved after integration and translated into measurable network-level changes rather than inferred from component-level sensitivity alone. This review develops a structure–response–application framework for multi-stimuli-responsive biomedical hydrogels. Strategies based on responsive polymer segments, dynamic covalent bonds, and functional nanocomposites are examined according to how responsive components are structurally integrated and coordinated to achieve application-specific functions. By connecting component integration with biomedical function, this cross-scale framework provides criteria for selecting integration strategies and assessing whether multiple responses are functionally coordinated rather than merely coincident.

## 1. Introduction

Hydrogels are three-dimensional crosslinked networks that absorb and retain large amounts of water while remaining insoluble in aqueous environments [1,2]. Their high water content, tissue-like characteristics, favourable biocompatibility, potential biodegradability, and tunable mechanical and transport properties have made hydrogels attractive materials for biomedical applications [3,4]. Advances in polymer chemistry and nanotechnology have further transformed these conventional hydrated materials into “smart” hydrogels through the incorporation of stimulus-sensitive components capable of recognizing changes in the surrounding environment [1,5]. The molecular responses of these components can subsequently be translated into macroscopic changes in hydrogel volume and phase behaviour, allowing material behaviour to be regulated by endogenous signals or externally applied stimuli [6]. Stimuli-responsive hydrogels therefore provide adaptive platforms that couple environmental recognition with changes in network structure and biomedical function.

**Figure 1:**
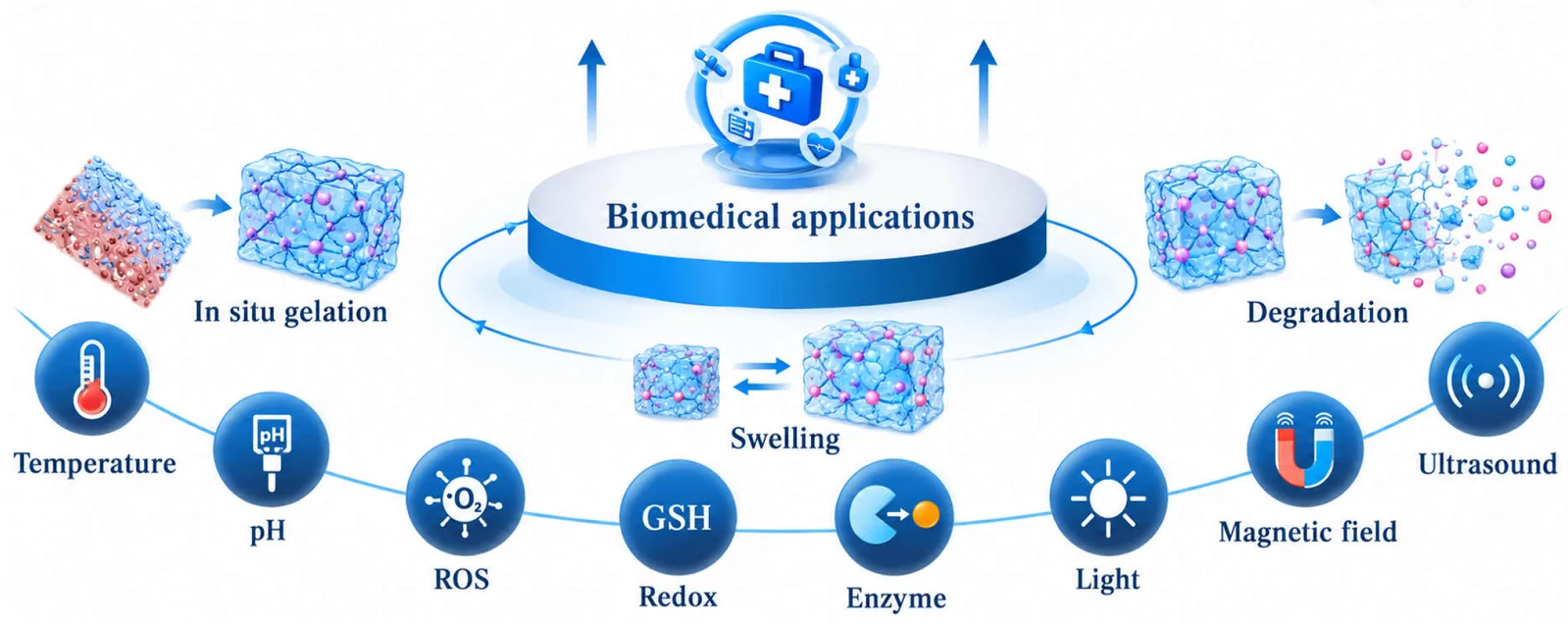
Schematic illustration of multi-stimuli-responsive hydrogels triggered by endogenous and exogenous stimuli for biomedical applications.

Disease-associated environments, however, generally present more than one pathological signal. Endogenous conditions, including pH, reactive oxygen species (ROS) levels, redox state, enzyme activity and glucose concentration, may vary across tissue regions, cellular environments, and different stages of disease progression [7,8,9]. An individual endogenous signal may also be insufficient in magnitude to reach the hydrogel activation threshold or too transient to maintain the resulting response throughout the intended treatment period. Moreover, hydrogels employed in drug delivery may encounter different signals sequentially along an administration route or during transport from extracellular environments into intracellular compartments, as exemplified by gastrointestinal and tumour-targeted delivery systems [10,11]. To improve activation selectivity and effectiveness under these conditions, multi-stimuli-responsive hydrogels have emerged as multifunctional platforms for addressing complex biomedical environments. By integrating multiple responses, these systems can coordinate network transformation with drug release or therapeutic activation (Figure 1). In diabetic wounds, glucose oxidase (GOX)-containing systems can convert elevated glucose into gluconic acid and hydrogen peroxide ($H_{2}O_{2}$), thereby coupling glucose recognition with subsequent pH- and ROS-mediated responses [12]. Hierarchical tumour-delivery systems have also combined acid-sensitive hydrogel networks with thermoresponsive micellar carriers, allowing the surrounding network and embedded drug reservoirs to regulate different stages of release [13].

External inputs, such as light, ultrasound, and magnetic fields, can further complement endogenous recognition by enabling remotely initiated and spatially or temporally controlled activation [14,15]. For example, near-infrared (NIR) photothermal conversion can be combined with endogenous pH responsiveness to coordinate external activation with pathological microenvironment recognition [16]. Ultrasound-generated ROS can similarly connect remotely applied acoustic energy with subsequent transformation of an oxidation-sensitive network [17]. Multiple external inputs can also be combined, as demonstrated by light- and magnetic-responsive hydrogels that provide complementary forms of remote regulation in wound treatment [18].

However, effective multi-stimuli-responsiveness depends not only on the incorporation of multiple responsive components but also on whether their response characteristics are retained after integration and whether stimulus-induced changes are effectively translated into the intended network-level behaviour. Recent reviews have established a valuable foundation by examining the response mechanisms, construction strategies, and therapeutic applications of composite stimuli-responsive hydrogels for wound-healing applications [3,4]. General synthesis strategies, individual response mechanisms, and biomedical applications of smart hydrogels have also been broadly surveyed [19]. Building on these contributions, the present review focuses specifically on systems with experimentally verified responses to multiple stimuli. The structural origin and integration of responsive components are systematically connected with their network-level expression. The coordination of individual responses is further examined within this unified framework. To ensure transparency in the evidence base, a structured literature search was conducted in PubMed and Web of Science for articles published between January 2020 and June 2026. The search combined hydrogel-related terms (“hydrogel” OR “gels”) with responsiveness-related terms such as “stimuli-responsive,” “multi-stimuli-responsive,” “dual-responsive,” “triple-responsive,” and “smart.” Specific stimulus terms, including “pH,” “temperature,” “ROS,” “redox,” “enzyme,” “glucose,” “light,” “ultrasound,” and “magnetic,” were also combined with the application term “biomedical application.” Earlier foundational studies were used only when necessary to explain established mechanisms or representative design strategies. Studies were included if they focused on a hydrogel-based system, experimentally demonstrated responsiveness to at least two stimuli, and investigated the construction strategy, response mechanism, or biomedical application of that system. Single-responsive systems were not included as primary examples of multi-responsive hydrogels. Nominally multi-responsive systems lacking experimental verification of multiple responses and studies with no biomedical relevance were excluded. Each included primary study was qualitatively evaluated according to material composition, integration strategy, stimulus conditions, experimental model, network-level response, and biomedical outcome.

On this basis, this review organizes multi-stimuli-responsive hydrogels according to the dominant structural origin of their responsiveness rather than solely according to stimulus type. Polymer-segment-based, dynamic-covalent-bond-based, and nanocomposite-based strategies are evaluated to determine how responsive components are integrated and how their molecular changes are translated into network-level variations in structure and molecular transport. Particular attention is given to how responsive components influence one another by shifting response windows, altering responsive-site accessibility, or modifying the network-level expression of individual responses. The independent, cooperative, sequential, and cascade coordination of different responses is also examined in relation to their functional roles in wound treatment, in vivo drug delivery, and tissue engineering systems. Collectively, this structure–response–application framework provides a basis for the rational design of multi-stimuli-responsive biomedical hydrogels.

## 2. Design and Construction Strategies of Multi-Stimuli-Responsive Hydrogels

Multi-stimuli-responsive hydrogels can be designed by assigning stimulus recognition, network regulation, and signal conversion to different structural components [20]. Responsive polymer segments incorporate stimulus sensitivity into the polymeric framework [21], whereas dynamic covalent bonds couple environmental changes directly to variations in network connectivity [22]. Functional nanomaterials serve primarily as energy transducers or field-responsive structural units, thereby extending hydrogel responsiveness to light, ultrasonic, and magnetic fields [23]. Catalytic nanomaterials further enable endogenous metabolites to be converted into chemical signals that initiate subsequent network or therapeutic responses [24]. Accordingly, this chapter discusses design strategies based on polymer segments, dynamic covalent bonds, and nanocomposites.

### 2.1. Design Based on Polymer Segments

Polymer segments serve not only as the structural constituents of the hydrogel network but also as functional modules for stimulus recognition and signal conversion [21]. In response to specific stimuli, reversible or irreversible molecular changes in these segments can be amplified into macroscopic variations in network structure and mechanical performance [25]. The resulting changes in network hydration, porosity, and association can further regulate molecular transport [26]. This section first summarizes the representative responsive polymer segments used in multi-stimuli-responsive hydrogels and then discusses the molecular- and network-level strategies employed to integrate them into hydrogel networks for achieving multi-stimuli-responsive behaviour.

#### 2.1.1. Individual Responsive Polymer Segments

Individual responsive polymer segments encompass intrinsically responsive polymers, functional groups grafted onto polymer backbones, and stimulus-labile linkages incorporated into polymer chains or network junctions. These segments recognize environmental or externally applied stimuli and may undergo one or more characteristic molecular events, including protonation or deprotonation, hydration or dehydration, conformational transitions, intermolecular association or dissociation, oxidation or reduction, and bond cleavage or exchange [27]. At the network scale, such molecular events may manifest as hydrogel swelling or contraction, sol–gel transitions, network degradation, and consequent changes in cargo diffusion or release kinetics [28]. In multi-stimuli-responsive hydrogels, individual segments may also regulate the accessibility of other responsive sites, amplify an initial response, or participate in sequential activation [10,29]. This section focuses on the responsive segments themselves and the molecular mechanisms underlying their functions in multi-responsive systems.

Thermoresponsive segments can be broadly divided into lower critical solution temperature (LCST)-type vinyl polymers, upper critical solution temperature (UCST)-type polymers, amphiphilic block copolymers, and naturally derived or bioengineered polymers. Among LCST-type polymers, poly(N-isopropylacrylamide) (PNIPAM) [18], poly[2-(dimethylamino)ethyl methacrylate] (PDMAEMA) [30,31], and poly(N-vinylcaprolactam) [32] undergo dehydration, chain collapse, and enhanced hydrophobic association upon heating. By contrast, UCST-type segments are stabilized by interchain associations below their transition temperatures and become increasingly hydrated as these associations dissociate upon heating. Representative systems include poly(N-acryloyl glycinamide) in temperature/NIR-responsive hydrogels [33] and the coiled-coil protein Q, whose UCST-type gelation is additionally coupled to pH-dependent fibrous assembly [34]. Amphiphilic poloxamers and methoxy poly(ethylene glycol) (mPEG)-based block copolymers undergo temperature-dependent micellization and subsequent gelation above a critical polymer concentration [20,35]. Methylcellulose [36], chitosan (CS)/β-glycerophosphate [16], gelatin [37], and elastin-like polypeptides (ELPs) [38] exhibit material-specific thermally induced association or conformational transitions. When incorporated into multi-responsive systems, thermoresponsive segments commonly enable injectable in situ gelation or regulate network dimensions and molecular transport, after which other responsive components can promote swelling, degradation, or release [39,40,41]. Photothermal or magnetothermal components can alternatively generate local heat to activate network contraction and cargo release [16,18]. However, the thermal response of the final hydrogel may differ substantially from that of the isolated thermoresponsive polymer, as demonstrated in several multi-responsive systems. Reliable thermoresponsive performance at the complete network composition requires comprehensive consideration of polymer concentration, hydrophilic–hydrophobic balance, and intercomponent interactions [32,42,43]. In the system examined, increasing the thermoresponsive polymer concentration promoted intermolecular association, lowered the sol–gel transition temperature, and accelerated gelation [32]. Chemical functionalization and interactions with charged components may further alter the onset and kinetics of thermal gelation [42,44].

Weakly acidic and basic groups provide the molecular basis for pH responsiveness, giving rise to anionic, cationic, and amphoteric polymer segments. Anionic polymers employed in multi-response hydrogels include poly(acrylic acid) (PAA) [25,45], poly(methacrylic acid) (PMAA) [46], carboxymethyl cellulose (CMC) [26], alginate [47], and succinoglycan (SG) [48]. Deprotonation increases the negative charge density, electrostatic repulsion, and hydration of these polymer chains, thereby generally promoting network swelling. Cationic polymers, including PDMAEMA [30], poly(L-lysine) (PLL) [49], and CS [50], exhibit the opposite ionization trend. Chemical modification can reverse or modify this behaviour: conversion of PLL into poly(L-lysine isophthalamide) (PLP) produces an anionic polymer that collapses through carboxyl protonation at low pH and expands following deprotonation [10,51]. Amphoteric poly(L-glutamic acid)/PLL networks undergo bidirectional changes in charge and association and can consequently respond to pH, ionic strength, and enzymatic degradation [52]. Polymers functionalized with pH-labile imine, hydrazone, or boronate ester groups provide a complementary route in which pH-dependent bond dissociation directly changes network connectivity. Because their pH sensitivity is governed primarily by bond-specific equilibria and exchange kinetics rather than by polymer-segment ionization alone [53], these systems are discussed separately in Section 2.2. Within multi-responsive networks, ionizable segments act as endogenous pH-recognition modules that regulate swelling, permeability, and the accessibility of other responsive sites [10]. Their effectiveness depends on whether the apparent pH-response window of the complete network remains aligned with the target microenvironment. This response window may be altered by chemical modification or interactions with other network components. Increasing the phenylboronic acid (PBA) grafting degree, for example, shifted and broadened the precipitation transition of PLP–PBA polymers [51]. Hydrophobic phenylalanine residues likewise raised the transition pH of polyglutamate networks, whereas electrostatic attraction between oppositely charged glutamate and lysine residues suppressed swelling over an intermediate pH range [54]. Consequently, the pH response observed at the network scale may differ from that expected for the isolated ionizable segment in both its effective window and the extent of swelling.

ROS- and redox-responsive segments respond either through oxidation-induced changes in polymer polarity or through cleavage of redox-labile bonds. Oxidation converts the hydrophobic thioethers in poly(propylene sulfide) and poly(L-methionine) (PMet) into more hydrophilic sulfoxide or sulfone groups [21,29]. This hydrophobic-to-hydrophilic transition can disrupt hydrophobic associations and promote micellar or polymer disassembly. Similar thioether responsiveness can be introduced by methionine modification of natural polymers [36]. Alternatively, oxidative cleavage of thioketal (TK) [55,56] or phenylboronate groups [22,57] and reductive cleavage of disulfides [10,58] directly reduce polymer molecular weight or network connectivity, whereas diselenide groups can respond under both oxidative and reductive conditions [59]. These units can function independently or participate in sequential, synergistic, or cascade pathways with other stimuli [17,60,61]. Their expression at the network scale is nevertheless governed by the local concentrations of ROS or reducing agents [62,63], as well as on the site accessibility of responsive segments [62]. Dense matrices may limit oxidant transport and coexisting non-responsive crosslinks can also prevent molecular cleavage from producing measurable degradation [29,55]. Conversely, enzyme- or catalyst-mediated ROS generation can amplify activation, although its efficiency depends on catalytic activity, oxygen availability, and light or ultrasound penetration [12,17,64]. Antioxidant components may consume ROS before it reaches the responsive groups [12,29]. An appropriate balance between ROS generation and network susceptibility is therefore essential to prevent insufficient activation, premature degradation, and undesired oxidative damage.

Enzyme responsiveness can be encoded either in sequence-defined peptide substrates or in naturally derived polymer backbones susceptible to enzymatic cleavage. Engineered peptide sequences provide specific enzyme-recognition sites whose cleavage disrupts polymer chains or network junctions, thereby inducing degradation and cargo release. Examples include a cyclic peptide linker cleavable by matrix metalloproteinases (MMPs) for infection-triggered antimicrobial peptide release [65], protease-sensitive self-assembling peptides degraded in wound environments [39], and poly(L-glutamic acid-co-L-lysine) degraded by intestinal trypsin [52]. In addition, naturally derived polymer backbones can themselves serve as enzymatic substrates, including hyaluronic acid (HA) for hyaluronidase [66], gelatin for MMPs [67], starch for amylase [68], and CS for lysozyme [69]. By exploiting specific enzyme–substrate recognition, these segments complement physicochemical responses with a biologically triggered degradation pathway in multi-responsive hydrogels. The resulting degradation rate is nevertheless controlled by local enzyme activity and substrate accessibility. Trypsin-mediated degradation of a pH- and enzyme-responsive polypeptide hydrogel was slower under acidic conditions [52]. In a separate system, genipin-crosslinked CS increased network stiffness while reducing the rate of proteinase K-mediated peptide degradation [39].

Photoresponsive segments are generally prepared by incorporating photoisomerizable, photodimerizable, or photocleavable groups into polymer backbones or side chains. Light drives reversible *trans–cis* isomerization of azobenzene (AB), altering its geometry, polarity, and affinity for cyclodextrin (CD) [70,71]. Reversible disruption and reformation of AB–CD junctions can therefore regulate network connectivity through host–guest interaction [72,73]. Spiropyran derivatives interconvert among the nonpolar, ring-closed spiropyran (SP), polar, ring-open merocyanine, and protonated merocyanine states in a manner dependent on irradiation wavelength, pH, and the surrounding polymer environment [74,75]. These changes in polarity and charge allow SP-functionalized polymers to couple light and pH responses with variations in swelling and molecular transport [74,76]. Coumarin- and anthracene-functionalized segments form additional covalent junctions through ultraviolet (UV)-induced photodimerization [69,77]. By contrast, *o*-nitrobenzyl and coumarin derivatives can undergo photocleavage to release conjugated cargo or disrupt network linkages [78,79]. Notably, photolysis of *o*-nitrobenzyl groups in a CMC hydrogel generated new nitrosothiol crosslinks and stiffened rather than degraded the network [78], while two-photon cleavage of coumarin-containing crosslinks under 808 nm irradiation promoted degradation and drug release [33]. Photoresponsive segments provide remote spatial and temporal control, but their macroscopic effects depend on the local chemical environment and the surrounding network architecture [74,75]. Network reinforcement may restrict AB–CD rearrangement [20,73], while the extent and reversibility of coumarin or anthracene crosslinking are governed by the photoreactive-group content and the irradiation conditions [69,77]. Molecular modification can shift activation toward visible light [70,80], whereas NIR activation usually requires two-photon excitation, upconversion, or energy-transfer pathways [71,80]. Since tissue attenuation reduces activation at depth and excessive irradiation may cause heating or photochemical damage, the wavelength and dose should be selected to balance tissue penetration, activation efficiency, and biological safety [81,82].

Although most responsive polymer segments are selected for their sensitivity to one dominant stimulus, some polymers inherently respond to multiple environmental signals. Representative examples include PDMAEMA, in which pH-dependent protonation is coupled to LCST-type thermoresponsiveness [30,83], and PMAA, whose carboxyl groups mediate both pH and ${\mathrm{Ca}}^{2+}$ responses [84]. CS combines protonation-dependent pH sensitivity with enzymatic degradability [85]. ELPs may integrate temperature responsiveness with pH or enzyme sensitivity [86], whereas protein Q couples UCST-type gelation with pH-dependent fibrous assembly [34]. Such polymers reduce the number of separately introduced components, but responses encoded within the same structure are often mechanistically coupled and difficult to tune independently. In PDMAEMA, pH-induced polymer protonation shifts its LCST [30]. The availability of carboxylates for ${\mathrm{Ca}}^{2+}$ in PMAA segments is also controlled by pH [84]. For natural polymers, additional stabilization may suppress an intrinsic response: crosslinking used to stabilize the gelatin network at physiological temperatures can restrict its thermoreversible rearrangement and enzymatic degradation [67,87]. The combined pH and hyaluronidase responses of HA are viable only when measurable ionization changes and sufficient enzyme activity occur within the same environment [66,88]. The selection of intrinsically multi-responsive segments should therefore consider whether their response windows overlap with the target conditions and whether the underlying molecular changes remain detectable in the complete network. Limited mechanical strength and stability, together with response windows that may not match physiological conditions, further motivate the integration of distinct responsive segments through the molecular- and network-level strategies discussed below.

#### 2.1.2. Molecular-Level Integration of Responsive Polymer Segments

Responsive polymer segments can be covalently integrated within the same polymer architecture through copolymerization or grafting. Copolymerization combines polymerizable monomers or macromolecular components with different responsive functions, whereas grafting introduces responsive segments or side groups onto existing polymer backbones. These approaches allow the type and relative content of responsive components to be adjusted before network formation. Table 1 summarizes representative molecular-level integration strategies, their responsive segments, response relationships, and biomedical applications.

Copolymerization provides a direct route for integrating polymerizable responsive units into the same polymer structure. In this approach, polymerizable monomers or macromolecular precursors are covalently connected through chain-growth or ring-opening polymerization, and the resulting copolymers can subsequently form hydrogels through chemical crosslinking or block-copolymer self-assembly. Temperature–pH copolymerization can combine thermally induced network transitions with ionization-dependent swelling or molecular retention. In PNIPAM hydrogels containing acidic comonomers, however, pH-dependent release remained limited at pH 6–8 because the carboxyl groups were already predominantly ionized [41]. In lignin-based poly(N-isopropylacrylamide-*co*-acrylic acid) [p(NIPAm-*co*-AAc)] networks, increasing the lignin methacrylate content from 0 to 16% reduced the equilibrium swelling ratio of the hydrogels from 24.81 to 2.20 g/g [89]. Within the testable range of 0–12.5%, the storage modulus ($G^{\prime }$) increased by up to approximately twofold. At 16%, however, the hydrogel was too brittle for tensile testing or reliable rheological measurements, illustrating the need to balance mechanical reinforcement against swelling capacity and network flexibility.

**Figure 2:**
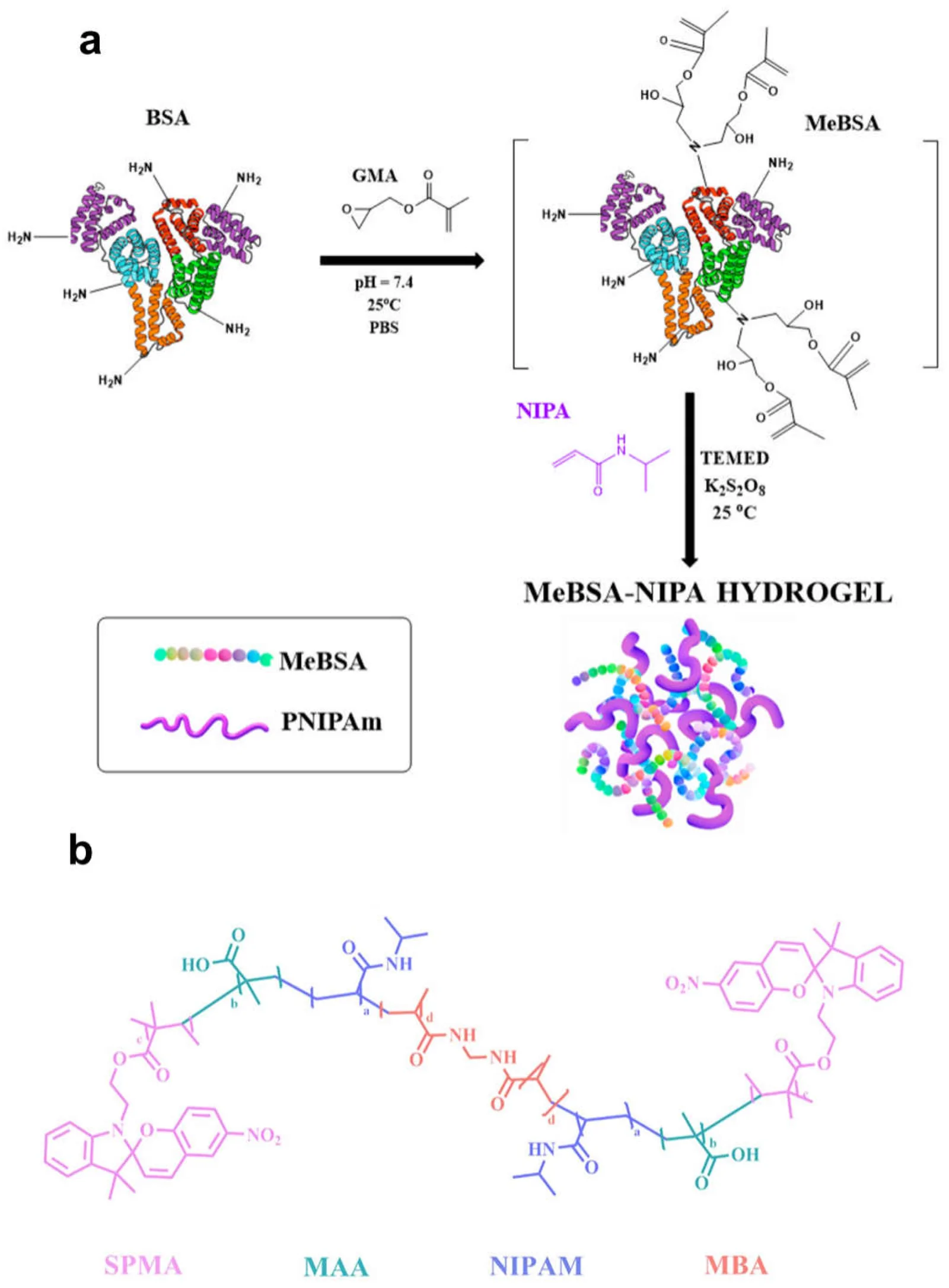
Schematic preparation and copolymer structures of (**a**) MeBSA–PNIPAM and (**b**) p(NIPAm-*co*-MAA-*co*-SPMA). (**a**) Reproduced from Ref. [43] under CC BY 4.0. (**b**) Reproduced with minor formatting modifications and permission from Cai et al. [76]. Copyright © 2025 Wiley Periodicals LLC.

The relative proportions of the copolymerized components provide a further means of regulating hydrogel responses and application-related properties. A dual pH- and temperature-responsive hydrogel was constructed by copolymerizing NIPAm with methacrylated bovine serum albumin (MeBSA) (Figure 2a). Increasing the MeBSA content introduced more polymerizable sites and ionizable amino acid residues, thereby altering the balance between network constraint and hydration [43]. These compositional changes shifted the volume phase transition temperature and determined how strongly the pH-dependent ionization and hydration of MeBSA affected the thermoresponsive contraction of PNIPAM. At MeBSA contents of 15% and 20%, the volume phase transition temperature decreased from 32.76 °C for pure PNIPAM to 27.04 and 28.88 °C, respectively. In contrast, the transition temperatures determined by swelling measurements ranged from 28.12 to 33.01 °C at pH 1.2 and from 29.07 to 33.64 °C at pH 7.4. These composition- and pH-dependent shifts show that copolymer composition can simultaneously regulate the response window and network-level response through changes in crosslink density, protein ionization, and network hydration.

In addition to conventional temperature–pH combinations, alternative responsive segments can be incorporated to diversify the stimuli recognized by the resulting copolymer. Ring-opening polymerization of L-methionine *N*-carboxyanhydride from a mPEG macroinitiator produced mPEG-*b*-PMet, which formed a thermoresponsive physical hydrogel through amphiphilic self-assembly [21]. Heating first promoted hydrophobic association and packing of the PMet-containing micelles, enabling in situ gelation at physiological temperature. Subsequently, ROS-mediated oxidation converted the methionine thioether groups into more hydrophilic oxidized groups, weakening these associations and accelerating hydrogel erosion and drug release. Increasing the H_2_O_2_ concentration from 1 to 10 mM shortened the time required for complete degradation of the hydrogel from 16 to 5 days, while more than 80% of DOX and resiquimod were released within 5 days at 10 mM H_2_O_2_. In vivo, the drug-loaded hydrogel suppressed tumour growth and reduced postoperative recurrence. The number of responsive modes can be further increased by incorporating additional polymerizable units. As shown in Figure 2b, free-radical copolymerization of NIPAm, methacrylic acid (MAA), and spiropyran methacrylate (SPMA), together with a covalent crosslinker, integrated temperature-, pH-, and UV-light-responsive units into a single p(NIPAm-*co*-MAA-*co*-SPMA) hydrogel [76]. Under low-temperature and acidic conditions, hydration of the NIPAm segments maintained an expanded network, while protonation of the MAA units weakened their electrostatic retention of the cationic drug. The combined thermal, acidic and UV stimulation conditions improved cumulative DOX release to approximately 89% within 6 h, which is a significant increase on the 67.87% observed under dual (thermal and acidic) stimulation [76]. The hydrogel also retained reversible swelling–deswelling behaviour over four alternating pH 2–12 cycles and four thermal cycles between 35 and 45 °C.

Copolymerization therefore allows the type, number, and relative content of responsive segments to be defined at the synthesis stage. Their responses may be coupled or operated sequentially under application-relevant conditions. However, the response of each segment in the resulting hydrogel is influenced by both its intrinsic properties and interactions among the copolymerized components. The response thresholds, magnitude, and kinetics of the resulting hydrogel should therefore be evaluated in relation to both copolymer composition and intersegment interactions within the complete network. Modification of an established copolymer system may also require adjustment of the monomer feed and resynthesis of the polymer or network. In comparison, grafting provides an alternative route for introducing additional responsive groups or adjusting the response characteristics of existing polymer backbones.

Post-polymerization functionalization provides a complementary route for introducing additional stimulus-sensitive moieties into existing polymer backbones without requiring complete redesign of their molecular architecture. It is applicable to both natural and synthetic polymers and can introduce a new stimulus-recognition pathway, regulate an existing response, or provide functional groups for subsequent network formation through targeted chemical modification.

A straightforward implementation is to graft a preformed responsive oligomer or polymer segment onto an established macromolecular platform [37]. Kang et al. grafted the pH-responsive OSM onto gelatin, allowing the ionization-dependent hydrophobic association of OSM to regulate thermally induced gelation [37]. Under mildly acidic conditions, reduced ionization of OSM strengthened the intermolecular association, thereby shifting the thermal-gelation window to a lower temperature. As the OSM/gelatin feed molar ratio increased from 10 to 30, the gelation temperature decreased from 33 to 27 °C at pH 7.4 and from 29 to 25 °C at pH 6.5. Increasing the OSM grafting level also raised the apparent p$K_{a}$ from 6.8 to 7.7–7.95, shifting the effective pH-response window toward physiological and tumour-associated conditions. Higher OSM grafting levels further increased precursor viscosity, reduced hydrogel porosity, and prolonged paclitaxel release. For the hydrogels prepared at OSM/gelatin feed molar ratios of 20 and 30, cumulative paclitaxel release after 9 days was 84% and 71% at pH 7.4, compared with 74% and 63% at pH 6.5, respectively. In orthotopic glioblastoma-bearing mice, the paclitaxel-loaded hydrogels markedly suppressed tumour progression.

The distribution of responsive functions between the backbone and grafted segments can also be reversed, with a pH-responsive macromolecular backbone serving as the platform for thermoresponsive grafts [26]. Madech et al. first synthesized mPEG-*b*-Poly(ε-caprolactone) (mPEG-*b*-PCL) block copolymers through ring-opening polymerization and subsequently grafted them onto CMC via carbodiimide-mediated coupling [26]. The resulting CMC-*g*-(mPEG-*b*-PCL) integrated the pH-responsive CMC backbone and thermoresponsive amphiphilic segments within a single graft copolymer. At higher pH, deprotonation of the CMC carboxyl groups enhanced interchain electrostatic repulsion and polymer–water interactions, maintaining the copolymer in the sol state and requiring a higher temperature for gelation. At lower pH, carboxyl protonation reduced electrostatic repulsion and strengthened hydrogen bonding and hydrophobic association, producing more compact chains and facilitating the conversion of thermally induced micellar packing into a continuous gel network. Accordingly, the gelation temperature increased from approximately 14 to 38 °C as the pH increased from 6.5 to 8.5. Increasing the PCL block length or polymer concentration further promoted hydrophobic association and micellar packing, thereby shifting gelation to lower temperatures [26]. These results show that grafting can not only provide additional responsiveness but also couple the molecular response of one component and compositional parameters to the transition window and network-level expression of another [26]. Besides introducing entirely new responsive segments, side-chain grafting provides finer control over the response of an existing polymer architecture. In tertiary-amine-modified mPEG–polypeptides, amine protonation and substituent hydrophobicity jointly regulated chain conformation, micellar association, and thermogelation [35]. More hydrophobic amines lowered the gelation temperature, whereas hydrophilic methylpiperazine groups maintained hydration and reduced high-temperature syneresis. The corresponding hydrogel released approximately 22% of the loaded DOX over 14 days at pH 7.0, compared with approximately 78% within 2 days at pH 6.5 and 81% within 12 h at pH 5.0. These results link side-chain chemistry with the pH-response window, thermal stability, and drug-release profile.

Overall, grafting enables responsive functions to be distributed between an established backbone and independently selected oligomeric, polymeric, or side-chain components. The examples discussed above show that the structure, and grafting level of these components can alter intermolecular association, thereby shifting the pH- and thermal-response windows and network formation. These effects depend not only on the intrinsic responsiveness of the grafted groups but also on grafting density, reaction-site accessibility, and the constraints imposed on the parent polymer. When separately prepared responsive polymers must retain greater structural independence, their integration can instead be achieved at the network level.

#### 2.1.3. Network-Level Integration of Responsive Polymer Segments

Network-level integration assembles independently prepared responsive polymer components within a common hydrogel architecture while retaining their distinct polymer structures. Their integration may involve physical junctions, topological interpenetration, or covalent connections within and between networks. These structural features often coexist within the same material. The following discussion examines representative network architectures in which such connections coordinate component-specific responses and translate them into macroscopic changes in hydrogel structure and function.

**Figure 3:**
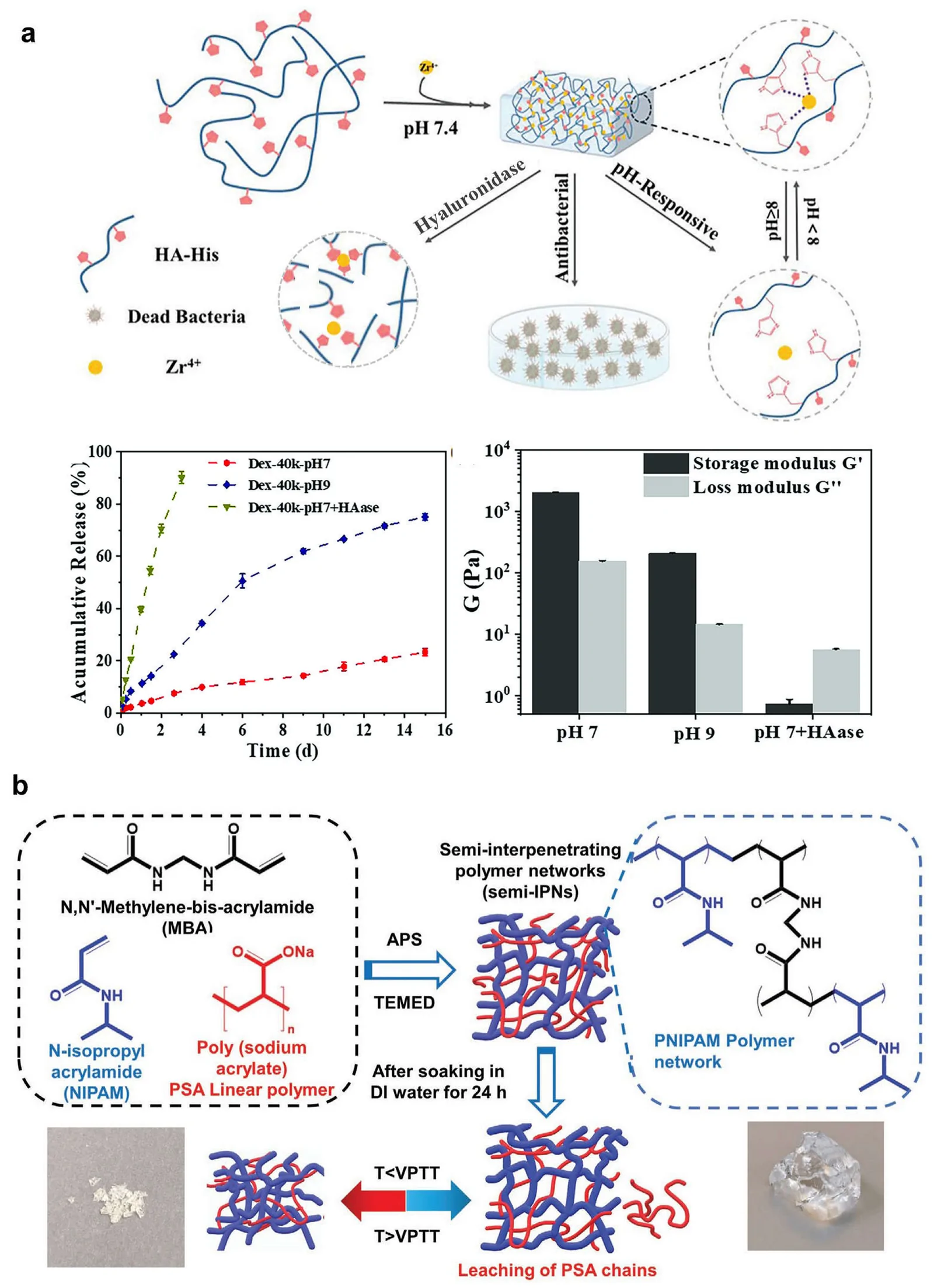
(**a**) Schematic illustration of the formation of the HA–His/Zr^4+^ hydrogel and its responsiveness to pH and hyaluronidase. The bottom panels show the cumulative release profiles of 40 kDa dextran from the HA–His/Zr^4+^ hydrogel under neutral (pH 7, red line), alkaline (pH 9, blue line), and hyaluronidase-containing (100 U/mL, pH 7, green line) conditions, together with the corresponding $G^{\prime }$ and $G^{\prime \prime }$ measured after 2 days of incubation under each condition. (**b**) Schematic fabrication of the semi-IPN hydrogel containing linear poly(sodium acrylate) (PSA) chains dispersed within the PNIPAM network. The lower part illustrates the leaching of PSA chains after the hydrogel was soaked in excess deionised water. Reproduced with permission from (**a**) Ref. [90], Copyright © 2022 Wiley-VCH GmbH, and (**b**) Ref. [91], Copyright © 2020 Wiley-VCH GmbH.

The construction of multi-stimuli-responsive hydrogels by physical integration involves the combination of different responsive polymers through non-covalent or supramolecular interactions. In these systems, stimulus-responsive behaviour may originate either from conformational or ionization changes in the polymer components themselves or from reversible association and dissociation of the physical junctions. Because of dynamic and reversible nature of noncovalent junctions, physically integrated networks are generally endowed with rapid stimulus responses, shear-thinning and self-healing behaviour [90]. As shown in Figure 3a, Zhou et al. developed physically crosslinked hydrogels via dynamic coordination between histamine-modified HA (HA-His) and ${\mathrm{Zr}}^{4+}$ ions [90]. The resulting metal–ligand networks exhibited pH-dependent mechanical changes and hyaluronidase-mediated degradation, together with pronounced shear-thinning behaviour and rapid self-healing. The storage modulus decreased from approximately 3.3 kPa at pH 5 to 2.0 kPa at pH 7 and to several tens of pascals at pH 8–9. In the presence of 100 U/mL hyaluronidase, the loss modulus ($G^{\prime \prime }$) exceeded $G^{\prime }$ after 2 days, indicating disintegration of the network. During repeated switching between 500% and 4% strain, the storage modulus fully recovered within seconds after each return to low strain, illustrating the rapid reorganization enabled by reversible noncovalent junctions. In a mouse full-thickness wound model, the hydrogel accelerated healing from day 3 to day 14 and promoted collagen deposition without evident systemic toxicity. However, networks supported by a limited number or type of physical junctions may exhibit insufficient mechanical strength and long-term stability [32].

Recent studies have shown that network stability can be improved by combining several noncovalent interactions or introducing components that provide additional physical associations [92]. For instance, a mechanically reinforced and self-healing luminescent hydrogel was achieved through the collaborative effect of hydrophobic association, terpyridine–${\mathrm{Eu}}^{3+}$ coordination, and electrostatic interactions [92]. Acidic protonation of terpyridine or hydroxide formation under alkaline conditions weakened the coordination crosslinks and promoted network swelling, decreasing the storage modulus from approximately 27 to 2 kPa. ${\mathrm{Fe}}^{3+}$ could independently access the same terpyridine sites and competitively displace ${\mathrm{Eu}}^{3+}$, resulting in luminescence quenching. The cooperation of these interactions reinforced the network while preserving the reversible junction dynamics required for self-healing. Nevertheless, increasing the number and strength of physical junctions may reduce their reversibility and restrict stimulus-induced network reorganization. The reversibility of physical junctions must therefore be balanced against network durability. Zhang et al. constructed a hydrogel from two bottlebrush copolymers with porphyrin- and oligoviologen-containing side chains, which were integrated through reversible host–guest interactions [93]. The density and accessibility of the recognition groups governed gelation and rheological properties: insufficient recognition groups resulted in inefficient gelation, whereas excessive hydrophilic spacer content hindered effective crosslinking and reduced the viscoelastic moduli. Electrostatic repulsion maintained the material as an injectable solution in deionised water, while charge screening in saline induced rapid gelation above approximately 20 mM NaCl. Heating and blue-light irradiation further reinforced the network by enhancing host–guest association and inducing a porphyrin-mediated photoredox process, respectively. In separate experiments, $G^{\prime }$ increased to approximately 470 kPa after heating at 80 °C for 10 min and to 400 Pa after 60 min of irradiation at 450 nm. The optimized hydrogel combined injectability and stimulus-induced reinforcement with maintenance of its gel state for at least four weeks in saline. This design strategy enabled the network to possess dynamic responsiveness, sufficient mechanical strength, and long-term stability simultaneously.

Greater structural stability can be achieved by topologically interpenetrating independently crosslinked responsive components. In an interpenetrating polymer network (IPN), two or more independently crosslinked polymer networks are mutually interpenetrated without being covalently bonded to one another. Topological entanglement can maintain component integration and suppress macrophase separation while allowing the constituent networks to retain their respective response mechanisms [94]. Bombonnel et al. sequentially confined separately crosslinked PAA and PNIPAM networks within the hydrophilic phase of a poly(butyl acrylate)-containing bicontinuous architecture [25]. Although the two responsive networks were not chemically connected to each other, their spatial interpenetration and confinement maintained their integration with increased storage modulus. Their pH- and temperature-response windows were preserved without measurable mutual interference.

However, the increased structural stability provided by an interpenetrating architecture may restrict the macroscopic expression of the individual network transitions. Topological confinement coupled the conformational changes of PAA and PNIPAM, such that PNIPAM contraction at elevated temperature limited the expansion of ionized PAA. Consequently, the initial release rate of acetylsalicylic acid at 42 °C and pH 7.8 was lower than expected from the simple combination of the two release-promoting responses. The bicontinuous architecture further slowed drug release by reducing the effective exchange surface and increasing the tortuosity of the diffusion pathways [25]. This effect was also demonstrated by physically interpenetrating gelatin/CS dual networks [87]. Introducing either an electrostatically crosslinked protonated CS network or a crystalline neutral CS network reduced the average pore size from 44.6 μm in the single-network hydrogel to 16.7 and 7.4 μm in the corresponding dual networks, respectively. The incorporation of these secondary CS networks also enhanced the mechanical stability of the overall hydrogels. The single-network hydrogel melted at 50 °C, whereas both dual networks remained in the gel state at the same temperature. This indicated that the secondary networks prevented the gelatin network from undergoing a gel–sol transition. Multifurcated vessels containing the crystalline neutral CS network maintained nearly unchanged stress–strain behaviour over ten cycles at 50% strain and preserved their tubular integrity in lysozyme-containing phosphate-buffered saline (PBS) at 37 °C for at least 3 months. The hydrogel also enabled the construction of free-standing multifurcated vessels that achieved endothelialization within 7 days. This example illustrates the trade-off between structural stabilization and macroscopic expression of individual responses.

Full IPNs therefore prioritize component retention and structural stability, although the crosslinking and topological confinement of both networks may attenuate their individual macroscopic responses. Semi-interpenetrating polymer networks (semi-IPNs) provide an intermediate architecture between direct polymer blending and full IPNs. In a semi-IPN, uncrosslinked linear chains are physically entangled within a crosslinked host network and may be further retained by noncovalent interactions. This architecture preserves greater conformational mobility than a full IPN while providing stronger structural confinement than direct polymer blending, allowing the linear chains to regulate swelling, pore structure, and mass transport in response to environmental changes. Hu et al. demonstrated this design using linear SG chains entangled within a crosslinked PNIPAM network [48]. The ionizable carboxyl groups of SG contributed pH-dependent swelling, while the PNIPAM network provided temperature responsiveness and structural stability. Increasing the SG content enhanced water uptake and flexibility while reducing the storage modulus from 535 to 185 Pa. At pH 7.4, the cumulative release from the hydrogel containing 30 wt% SG increased from approximately 77% at 25 °C to 92% at 37 °C within 36 h. However, the greater mobility that facilitates responsiveness also poses a retention risk: insufficiently confined linear chains may gradually diffuse or leach from the network during long-term use [91]. Through quantitative modulation of synthesis parameters of PNIPAM/PSA semi-IPNs, Gupta et al. found that the retention of linear PSA strongly depends on its molecular weight and polymerization temperature (Figure 3b) [91]. The resulting hydrogel properties integrated through physical associations and topological interpenetration depend not only on balancing the reversibility and strength of physical junctions, but also on the degree of topological interpenetration. Stronger physical associations or confinement improves structural stability but may restrict network reorganization or reduce the macroscopic expression of individual responses. Additionally, insufficient integration may result in weakening of the network or diffusion of uncrosslinked components.

Permanent covalent integration links responsive polymer components within a common covalently crosslinked network or introduces covalent bridges between otherwise distinct interpenetrating networks. Because these covalent junctions remain intact during stimulation, changes in swelling and volume arise primarily from conformational or ionization transitions of the constituent polymers rather than by cleavage of the network. This approach can improve component retention and structural stability during repeated stimulation. In these systems, the response magnitude and structural stability are governed by polymer composition and crosslink density and can be further influenced by how the responsive components are connected within the network [50,95].

Thiol–ene chemistry provides a versatile route for covalently integrating pre-functionalized responsive macromers into a common network. Ding et al. combined allyl-modified CS (OAL-CS) with dithiol-terminated PNIPAM (PNIPAM-SH) under UV irradiation to produce a pH- and temperature-responsive hydrogel [50]. The two polymer components retained their respective responses, while variation of the OAL-CS/PNIPAM-SH ratio regulated the crosslink density and swelling behaviour. The PNIPAM component exhibited a LCST of approximately 32 °C, while CS protonation produced the highest swelling at pH 5.0–6.0 and the lowest swelling at pH 9.0. Increasing the PNIPAM-SH content increased the crosslink density, leading to a stiffer network with a lower swelling ratio. Conversely, a lower PNIPAM-SH content resulted in a less densely crosslinked network, enabling more pronounced pH- and temperature-dependent swelling responses. Across the tested ranges of pH 5.0–9.0 and 4–45 °C, the swelling ratio varied by up to approximately 22-fold.

Permanent covalent bridges can also be introduced between interpenetrating responsive networks to improve their durability during repeated swelling and contraction. A crosslinked-network IPN hydrogel was established by Wiwatsamphan and Chirachanchai, using a combination of CS and PNIPAM networks via thiol–ene bonds [95]. The resulting hydrogel repeatedly changed between approximately 20% swelling at 10 °C and 40% shrinkage at 40 °C over 20 cycles and preserved its original size and shape for 28 days in the acidic buffer (pH 3.6). In contrast, the corresponding IPN without inter-network covalent connections began to crack after four to five stimulus cycles and lost its shape within one day under the same acidic conditions. Compared with the CS/PNIPAM IPN without inter-network covalent connections, the covalently coupled hydrogel exhibited smaller dimensional changes during temperature cycling. The additional covalent bridges therefore improved structural stability but reduced the stimulus-induced dimensional changes [95]. Permanent co-crosslinking and inter-network covalent coupling improve component retention and resistance to repeated deformation, although additional crosslinks may restrict swelling and dimensional responses.

Permanent covalent networks can also be endowed with degradability by incorporating stimulus-cleavable bonds into otherwise stable crosslinkers. Ali et al. crosslinked norbornene-modified CMC with a tetrazine-terminated TK linker through an inverse electron-demand Diels–Alder click reaction [61]. The network remained stable without stimulation, whereas NIR irradiation promoted indocyanine-green-mediated ROS generation and cleavage of the TK groups, leading to network de-crosslinking and accelerated drug release. Without stimulation, mass loss remained below 19% after 40 days and DOX release reached approximately 25% after 168 h. NIR irradiation for 20 min increased these values to approximately 60% and 96%, respectively. The loaded hydrogel thus exhibited enhanced antitumour activity under NIR irradiation. However, this stimulus-induced cleavage is irreversible, causing a permanent loss of network connectivity after activation. Although this one-way degradation is advantageous for on-demand drug release, it prevents reconstruction of the original network and limits repeated stimulus responses and structural recovery. By contrast, dynamic covalent junctions can undergo reversible exchange or dissociation–reformation reactions, allowing the network to reorganize or recover while maintaining covalent integration of the polymer components.

Network-level integration combines independently prepared responsive polymer components through physical associations, topological interpenetration, or permanent covalent connections. These architectures provide increasing component retention and structural fixation but progressively restrict network reconfiguration. Stimulus-cleavable covalent junctions can additionally introduce triggered disassembly, whereas dynamic covalent bonds combine covalent connectivity with reversible network rearrangement and are discussed separately below.

### 2.2. Design Based on Dynamic Covalent Bonds

**Figure 4:**
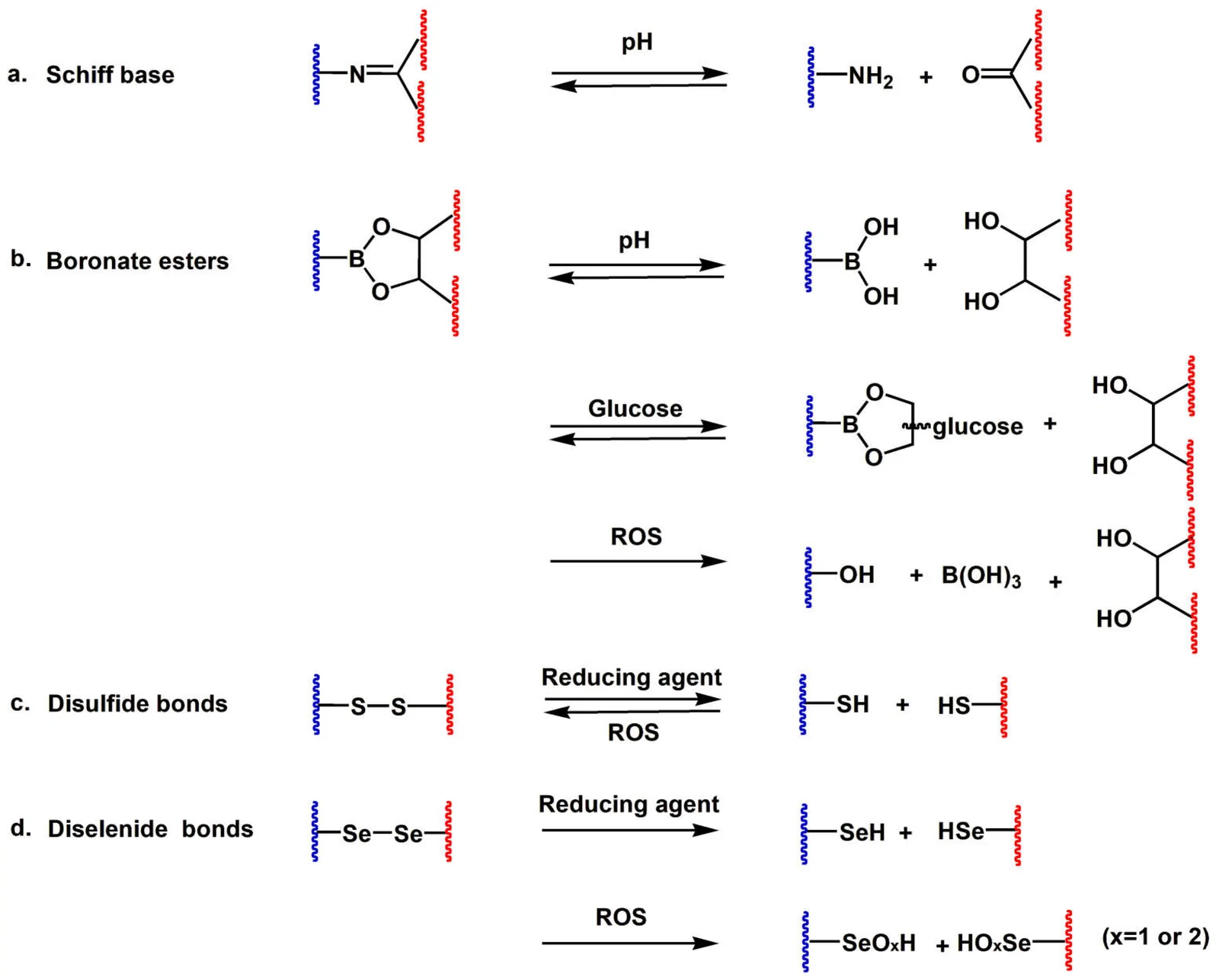
Representative stimulus-induced transformations of dynamic covalent bonds in multi-stimuli-responsive hydrogels: (**a**) pH-responsive Schiff-base hydrolysis; (**b**) pH-, glucose-, and ROS-responsive transformations of boronate esters; (**c**) redox interconversion between disulfide bonds and thiols; and (**d**) reductive and oxidative transformations of diselenide bonds. Wavy lines denote polymer chains.

Dynamic covalent bonds are a special class of covalent linkages that can undergo reversible cleavage and reformation or direct bond exchange in response to applied endogenous or externally applied stimuli [96]. When introduced as crosslinks into polymer networks, these bonds combine the structural integrity of covalent hydrogels with the adaptive characteristics commonly associated with reversible networks [96,97]. Their reversible rearrangement allows covalent network connectivity to be reorganized after gel formation, thereby supporting stress relaxation and network repair while generally providing more persistent network connectivity than rapidly exchanging physical junctions [97]. More importantly, dynamic covalent chemistry provides a versatile platform for constructing multi-stimuli-responsive hydrogels. A single dynamic linkage may be regulated by more than one environmental factor, whereas different dynamic bonds can be incorporated into the same network and activated independently, cooperatively, or sequentially [98]. These bonds can also be coupled with intrinsically responsive polymer segments or other functional components, thereby retaining the inherent responsiveness of the polymer backbone while introducing additional response pathways. Dynamic covalent networks therefore offer a programmable means of integrating multiple stimulus–bond–function relationships while maintaining a balance between network stability and adaptive responsiveness. Common dynamic covalent bonds employed in multi-responsive hydrogels include Schiff-base linkages, such as imines, hydrazones, and acylhydrazones, as well as boronate esters, disulfides, and diselenides. Schiff-base linkages are predominantly pH-responsive [53,99], boronate esters can respond to pH, glucose, and ROS [22,57], and disulfide or diselenide linkages are primarily regulated by redox conditions [40,59]. The representative stimulus-induced transformations of these dynamic covalent bonds and their resulting products are summarized in Figure 4.

Beyond their stimulus responsiveness, these bonds also offer unique advantages for biomedical applications. Natural polysaccharides are widely used as precursors because their amino and diol groups can participate in dynamic crosslinking, whereas their carboxyl groups provide convenient sites for introducing boronic acid, hydrazide, or thiol functionalities [44,53,66]. Macromolecular precursors containing complementary reactive groups allow dynamic networks to be formed without introducing an additional low-molecular-weight crosslinker while retaining the biological or enzymatic properties of the parent polymers [53,66]. Redox-responsive disulfide networks can likewise be constructed by conjugating thiol-containing molecules to polysaccharide backbones [44] or by directly using disulfide-containing poly(ethylene glycol) (PEG) derivatives [100,101]. In addition to connecting polymer precursors, dynamic covalent bonds can also combine network formation with covalent drug immobilization. Participation of the therapeutic agent in crosslinking can reduce its premature diffusion, whereas stimulus-induced bond dissociation enables its subsequent release. Representative examples include therapeutic polyphenols incorporated as boronate ester crosslinkers [22,102] and tobramycin (TOB) integrated through pH-labile Schiff-base linkages [103]. According to the dominant structural origin of multi-stimuli-responsiveness, these systems can be divided into three principal categories: single dynamic bonds responsive to multiple stimuli, integration of dynamic covalent bonds with other responsive components, and hybrid networks containing multiple dynamic covalent linkages.

#### 2.2.1. Single Dynamic Bonds with Intrinsic Multi-Stimuli-Responsiveness

Although multi-responsive hydrogels are commonly constructed by combining several responsive motifs, some dynamic covalent bonds can be regulated by more than one stimulus. In these systems, different stimuli regulate the same type of dynamic junction, enabling multi-responsive network changes through a single crosslinking chemistry.

Among dynamic covalent linkages, PBA-derived boronate esters are particularly attractive because a single boronic acid–diol binding motif can respond to pH, glucose, and ROS [22]. Acidic conditions shift the boronate ester equilibrium toward the free boronic acid and diol, whereas glucose competitively binds to the boronic acid groups and reversibly displaces polymer-bound diols. Instead, $H_{2}O_{2}$ oxidizes the arylboronic acid or ester into phenolic products, irreversibly consuming the responsive functionality. Thus, pH and glucose regulate reversible hydrolysis and competitive exchange, whereas ROS oxidation provides an irreversible pathway for network de-crosslinking. The response window of PBA-derived crosslinks can be further adjusted through the molecular structure and local environment of the boronic acid groups. The response window can be shifted toward mildly acidic or near-neutral conditions by lowering the ${\mathrm{pK}}_{a}$ of the boronic acid through electron-withdrawing substituents, neighboring-group interactions, or heteroaryl structures [104]. These structural modifications are important because the stability and glucose sensitivity of the resulting hydrogel depend strongly on whether the boronic acid–boronate equilibrium falls within the pH range of the target biological environment.

The combination of responsiveness to pH, glucose and ROS on a single boronate ester crosslink is particularly relevant to diabetic wounds, where elevated levels of glucose and ROS are accompanied by variable local pH levels over time and space [98]. Liu et al. demonstrated this single-bond–triple-response strategy by crosslinking PBA-functionalized sodium hyaluronate with tannic acid (TA) through dynamic boronate ester junctions [22]. The same crosslinks dissociated more rapidly under acidic conditions, elevated glucose concentrations, or $H_{2}O_{2}$ exposure, promoting network disruption and TA release. Notably, this dynamic network also displayed rapid self-healing and mechanical fatigue resistance, as evidenced by the recovery of gel-like behaviour over three alternating 0.3–100% strain cycles. Because TA simultaneously served as a crosslinker and an antioxidant therapeutic component, bond dissociation directly coupled pathological stimulus recognition with ROS scavenging and diabetic wound treatment [22]. Nevertheless, the acidic response observed at pH 5.0 should be interpreted as evidence of bond sensitivity rather than as a universally representative pH condition for diabetic wounds, whose pH varies with wound type and healing stage. The same principle has been applied to glucose-regulated insulin delivery. Loeffelsend and Groll functionalized three-arm star-shaped poly(2-methyloxazoline) with a nitro-substituted phenylboronic acid and crosslinked it with poly(vinyl alcohol) (PVA) to form injectable and self-healing hydrogels [105]. The boronic acid derivative had an apparent p$K_{a}$ of approximately 6.9, enabling hydrogel formation and maintaining stability near physiological pH. In addition to their self-healing ability, these hydrogels demonstrated good fatigue resistance, maintaining stable recovery of $G^{\prime }$ and $G^{\prime \prime }$ over repeated 0.01–100% strain cycles. The hydrogels exhibited glucose-dependent insulin release: increasing glucose concentrations progressively disrupted the boronate ester junctions through competitive binding and thereby accelerated insulin release. The glucose response was evaluated at concentrations of 1.0–12 mg/mL, with insulin release increasing from 57% to 98% after 300 min. However, the highest concentration represented a high-concentration challenge condition. Meanwhile, changes in pH altered the boronate ester equilibrium and consequently regulated network stiffness [105]. This mechanical response was examined at pH 1, 3, 5, 7, and 11, with only the near-neutral condition approximating the subcutaneous environment. Although network formation was supported near physiological pH, glucose-dependent network and release responses were not separately quantified at pH 7.4. These results demonstrate that the same dynamic linkage can translate pH and glucose variations into distinguishable changes in hydrogel mechanics and molecular release, although their combined operation at physiological pH requires more direct quantitative validation.

Cyclic hemiaminals represent an emerging but incompletely validated route to single-motif multi-responsiveness. In a recent study, semiclosed eight-membered cyclic hemiaminal junctions were formed by condensation between poly(ethylene glycol) diamines and polyformaldehyde, with the cyclic structures functioning as both network crosslinks and formaldehyde reservoirs [106]. The resulting hydrogel also exhibited rapid self-healing and mechanical durability, with $G^{\prime }$ and $G^{\prime \prime }$ recovering over repeated 1–2000% strain cycles. Acid-triggered degradation and formaldehyde release were directly demonstrated in the complete cyclic hemiaminal-crosslinked hydrogel. However, glutathione (GSH)- and H_2_O_2_-induced degradation was examined mainly using a model cyclic hemiaminal compound exposed to 100 mM GSH or H_2_O_2_. Although ROS scavenging by the complete hydrogel was demonstrated in vitro and in diabetic wounds, this finding does not directly establish H_2_O_2_-induced network de-crosslinking. This system is therefore best regarded as a promising candidate for equilibrium-coupled multi-responsiveness rather than a fully validated platform for pH, GSH, and H_2_O_2_ responses in the complete network.

Disulfide and diselenide bonds constitute another important group of dynamic linkages that can respond to more than one stimulus. Disulfide bonds predominantly function as reduction-responsive linkages: reducing agents such as GSH or dithiothreitol (DTT) cleave the S–S bonds into thiols, thereby releasing conjugated cargos or decreasing network connectivity [40,78]. Oxidation of the resulting thiols may regenerate disulfide bonds during network formation or recovery [78]. By contrast, diselenide bonds are more readily transformed under both reductive and oxidative conditions, owing to the lower bond energy and higher polarizability of Se–Se bonds. In a representative hydrogel, the same Se–Se crosslinks were disrupted by DTT and $H_{2}O_{2}$, accelerating hydrogel de-crosslinking and DOX release [59]. Although the NIR responsiveness is mediated by a separate photosensitizing component rather than by direct NIR activation of the Se–Se bond, this system directly verifies the dual oxidation/reduction responsiveness of a common diselenide crosslink [59].

In addition to chemical redox reactions, disulfide and diselenide bonds are susceptible to certain physical energy. Typically, UV irradiation can induce disulfide photolysis and thiyl-radical formation, particularly in strained cyclic disulfides [100,107]. The weaker Se–Se bond can undergo radical-mediated exchange under visible light or heating [108] and mechanochemical cleavage under sonication or osmotic pressure has also been demonstrated in diselenide-containing polymer systems [109,110]. Nevertheless, these physical response pathways have not all been systematically verified in complete hydrogel networks under biomedical conditions.

Overall, single dynamic bonds can integrate multiple stimuli through either different equilibrium involving the same linkage or distinct cleavage and exchange pathways. Boronate esters currently provide the most clearly established example that different stimuli act on the same crosslinking motif through distinct reversible or irreversible pathways. Cyclic hemiaminals represent an emerging candidate for equilibrium-coupled multi-responsiveness, whereas diselenide bonds provide distinct reductive and oxidative response pathways. For emerging dynamic motifs, each proposed response pathway must be verified in the complete hydrogel rather than inferred from a model compound or isolated linkage. In particular, responses mediated by photosensitizers, thermoresponsive polymers, or other functional components should not be attributed solely to the dynamic bond and are discussed in the following section on the integration of dynamic covalent bonds with responsive components.

#### 2.2.2. Integration of Dynamic Covalent Bonds with Other Responsive Components

Dynamic covalent crosslinks can be integrated with responsive polymer backbones or other functional components so that different stimuli act at distinct structural levels of the hydrogel. In such systems, the dynamic bonds regulate crosslink connectivity, whereas the connected components provide complementary responses through backbone degradation, conformational transitions, or changes in supramolecular organization. This strategy preserves the inherent responsiveness of the constituent materials while providing bond-level control over network stability and disassembly.

Natural polymers that retain susceptibility to enzymatic degradation provide representative examples of this strategy. Wang et al. crosslinked 3-amino PBA-functionalized HA with the diol-containing polyphenols of a natural plant extract through pH-responsive boronate ester junctions [66]. Acid-induced dissociation of the dynamic junctions and hyaluronidase-mediated cleavage of the HA backbone acted at the crosslink and polymer-backbone levels, respectively, to accelerate network disruption and silver nanoparticle (AgNP) release [66]. This concept has also been extended beyond HA-based networks. Beaman et al. prepared a shape memory polymer hydrogel foam by PVA and cornstarch using a disulfide-containing polyurethane crosslinker [68]. Amylase selectively degraded the starch component, whereas DTT cleaved the disulfide-containing crosslinker. Combined treatment with amylase and the reducing agent accelerated the degradation of starch-containing hydrogels, demonstrating complementary regulation at the polymer-component and crosslink levels [68].

Dynamic covalent bonds can also regulate the confinement and release of responsive carriers, thereby coordinating network- and carrier-level transitions. A boronate ester network first released acid-sensitive micelles under inflammatory conditions, after which micellar dissociation sustained drug release [111]. In another system, NIR-induced heating enhanced transport through thermoresponsive NP coatings while photogenerated ROS cleaved diselenide network junctions [112]. Reduction-labile covalent linkages can be structurally integrated with photoresponsive host–guest associations to form shared supramolecular junctions. Kim et al. constructed a supramolecular hydrogel by combining AB-grafted CMC chains with disulfide-linked CD dimers, whose two CD cavities enabled host–guest bridging between different polymer chains [73]. Light-induced AB isomerization weakened the AB–CD associations, whereas reductive cleavage of the disulfide bridge converted the bifunctional CD dimers into monofunctional units with reduced network connectivity. The two stimuli therefore disrupted different components of the same structurally coupled junction, providing complementary pathways for network disassembly and drug release [73]. Mechanical cycling tests further revealed that the hydrogel could repeatedly recover its network structure under alternating 0.5% and 900% strain, with a self-healing efficiency of 81.59% after three cycles.

Collectively, these systems demonstrate that dynamic covalent junctions can thus be coordinated with responsive polymer backbones, assemblies, nanocarriers, and supramolecular interactions to regulate network disassembly and therapeutic release across multiple structural levels.

#### 2.2.3. Hybrid Dynamic Covalent Networks for Multi-Stimuli Integration

More complex multi-stimuli-responsiveness can be achieved by incorporating multiple dynamic covalent linkages into the same hydrogel network. This strategy allows distinct responsive linkages to act in a coordinated manner within complex pathological microenvironments. Depending on the selectivity, temporal relationship, and network-level consequences of their activation, these systems can exhibit selective, parallel cooperative, or sequential response modes.

In selectively activated networks, different stimuli preferentially regulate distinct dynamic bonds, thereby controlling corresponding structural or therapeutic outputs with limited mutual interference. The defining feature is therefore the specificity of the stimulus–bond–function relationship rather than complete mechanical independence of the different crosslinks. For example, Wang et al. constructed a dual-dynamic network from PBA-modified gelatin and oxidized pullulan, with epigallocatechin-3-gallate (EGCG) and TOB incorporated as antioxidant and antibacterial agents, respectively [103]. In this system, Schiff base bonds immobilized TOB within the network, whereas phenylboronate ester bonds connected EGCG and other diol-containing components to the PBA groups. Acidification preferentially promoted Schiff base hydrolysis and TOB release, while elevated $H_{2}O_{2}$ oxidized the phenylboronate ester junctions and accelerated EGCG release [103]. Importantly, the two pathways showed limited cross-interference—TOB release was only marginally affected by increasing $H_{2}O_{2}$ concentration, and EGCG release remained largely insensitive to acidic conditions in the absence of additional ROS [103]. This architecture allowed acidic and oxidative signals to regulate complementary therapeutic functions through different dynamic junctions within the same network.

In parallel cooperative systems, multiple dynamic bonds are activated over a similar period by coexisting pathological signals, and their combined disruption produces a more pronounced network-level response than activation of either linkage alone. The participating bonds may remain chemically orthogonal at the molecular level while acting cooperatively to reduce overall network connectivity. Liu et al. developed an injectable hydrogel crosslinked through pH-labile imine bonds and reduction-labile disulfide bonds [58]. The hydrogel exhibited rapid self-healing and mechanical durability, showing reversible solid–fluid transitions with stable recovery over repeated 1–400% strain cycles. In the acidic and GSH-rich tumour microenvironment, imine hydrolysis and disulfide reduction occurred through distinct reaction pathways but jointly accelerated network disintegration and the release of paclitaxel and curcumin. The coordinated release enhanced tumour inhibition, and curcumin mitigated paclitaxel-associated inflammation [58]. This system illustrates how chemically selective dynamic bonds can be activated in parallel to generate a cooperative material and therapeutic response.

**Figure 5:**
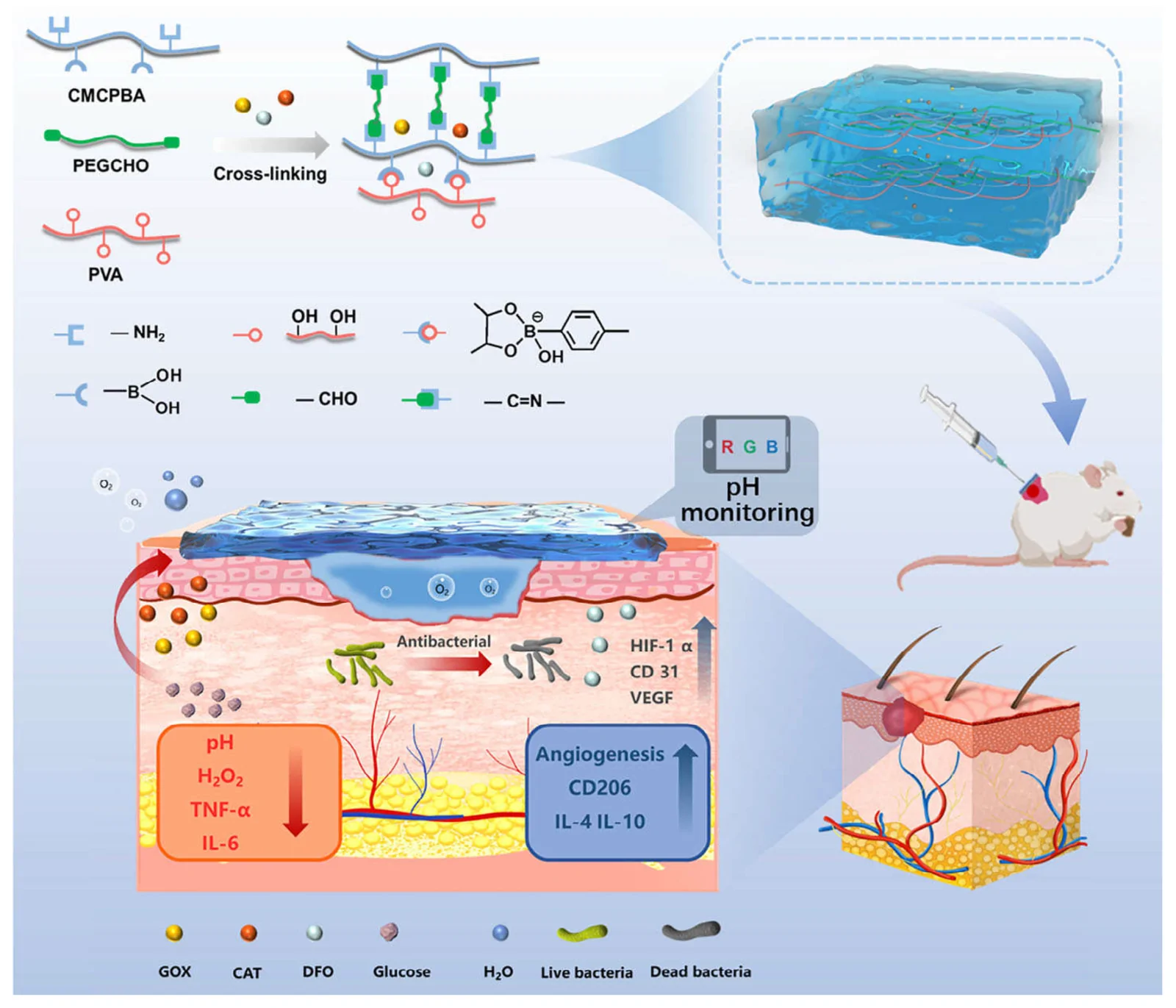
Schematic illustration of hydrogel formation through phenylboronate ester and Schiff-base crosslinks and the proposed glucose-triggered cascade regulation of the diabetic wound microenvironment. The temporal order of boronate ester disruption and Schiff-base hydrolysis was inferred from the reaction pathway and was not directly resolved by time-dependent analysis. Reproduced with permission from Ref. [12]. Copyright © 2025 Wiley-VCH GmbH.

Sequential systems introduce a causal relationship between different response pathways. The first stimulus either disrupts an initial junction, alters bond accessibility, or is converted into a secondary chemical signal that subsequently activates another dynamic bond. Such systems differ from parallel activation because the later response depends on an event generated by the earlier one. To achieve cascade-mediated sequential responsiveness, Zhou et al. designed a diabetic wound hydrogel containing glucose-responsive phenylboronate ester junctions and acid-labile Schiff base crosslinks, together with GOX and catalase (CAT), as shown in Figure 5 [12]. Glucose directly competed with polymer-bound diols for the PBA groups, thereby loosening the boronate ester network. In parallel, GOX converted glucose into gluconic acid and $H_{2}O_{2}$. The resulting acidification subsequently promoted Schiff base hydrolysis, while CAT decomposed $H_{2}O_{2}$ to limit oxidative accumulation and generate oxygen [12]. Through this enzyme-mediated signal conversion, the initial glucose input was coupled to both direct disruption of the boronate ester junctions and secondary pH-dependent destabilization of the Schiff base crosslinks, promoting drug release and remodeling of the diabetic wound microenvironment. Although this mechanism constitutes a rationally designed cascade, the temporal sequence of the two bond-cleavage events was inferred from the reaction pathway rather than directly resolved through time-dependent analysis of each crosslink.

Hybrid dynamic covalent networks can produce selectively activated, parallel cooperative, or sequential responses, depending on which bonds are affected and how their disruption changes network connectivity. Because these modes may overlap, their classification should be based on experimentally demonstrated stimulus–bond relationships and network consequences rather than on bond composition alone. To apply this criterion consistently, Table 2 compares representative dynamic-covalent systems in terms of bond-associated activation conditions, other responsive mechanisms, key network parameters, release performance, and biological outcomes. A response mode is recorded only when a selective, cooperative, sequential, cascade, independent, or self-regulating relationship was explicitly proposed in the original study. The level of resolution further distinguishes relationships supported by cross-stimulus, combined, simultaneous, sequential, or repeated-cycle experiments from cases in which multiple responses were demonstrated but their interaction remained unresolved. This comparison shows that only a limited number of studies have experimentally resolved the relationships between individual responses, whereas most have primarily established multi-stimuli responsiveness.

### 2.3. Design Based on Functional Nanomaterials

Functional nanocomposite modification expands the design space of multi-stimuli-responsive hydrogels by introducing nanoscale components capable of converting external energy into heat, catalyzing endogenous substrates, or undergoing field-directed reorganization [23,72]. Nanomaterials may act as active signal transducers to convert light, ultrasound, or magnetic energy into heat or reactive species [15,113]. A complementary mechanism relies on catalytic activity to convert endogenous substrates into chemical signals that remodel pathological microenvironments, cleave stimulus-labile bonds, or trigger therapeutic release [24,56]. Beyond signal conversion, surface-functionalized nanomaterials may also participate in hydrogel construction as physical or covalent network junctions, improving particle retention, mechanical stability, or structural organization [15,23]. The following discussion is organized according to the dominant response function of the nanomaterials: photothermal conversion, catalytic and ultrasound activation, and magnetic regulation.

#### 2.3.1. Photothermal-Responsive Nanomaterials

Photothermal nanomaterials used in multi-stimuli-responsive hydrogels mainly include inorganic nanomaterials, carbon nanostructures, and organic photothermal agents such as polydopamine (PDA). By converting light—particularly NIR light—into localized heat, they can regulate gelation, reversible network transitions, therapeutic release, as well as local hyperthermia for therapeutic applications. These materials may be retained within a hydrogel to introduce photothermal activity. Their surface groups or interfacial interactions may also contribute to network construction, thereby improving nanomaterial retention and hydrogel stability.

**Figure 6:**
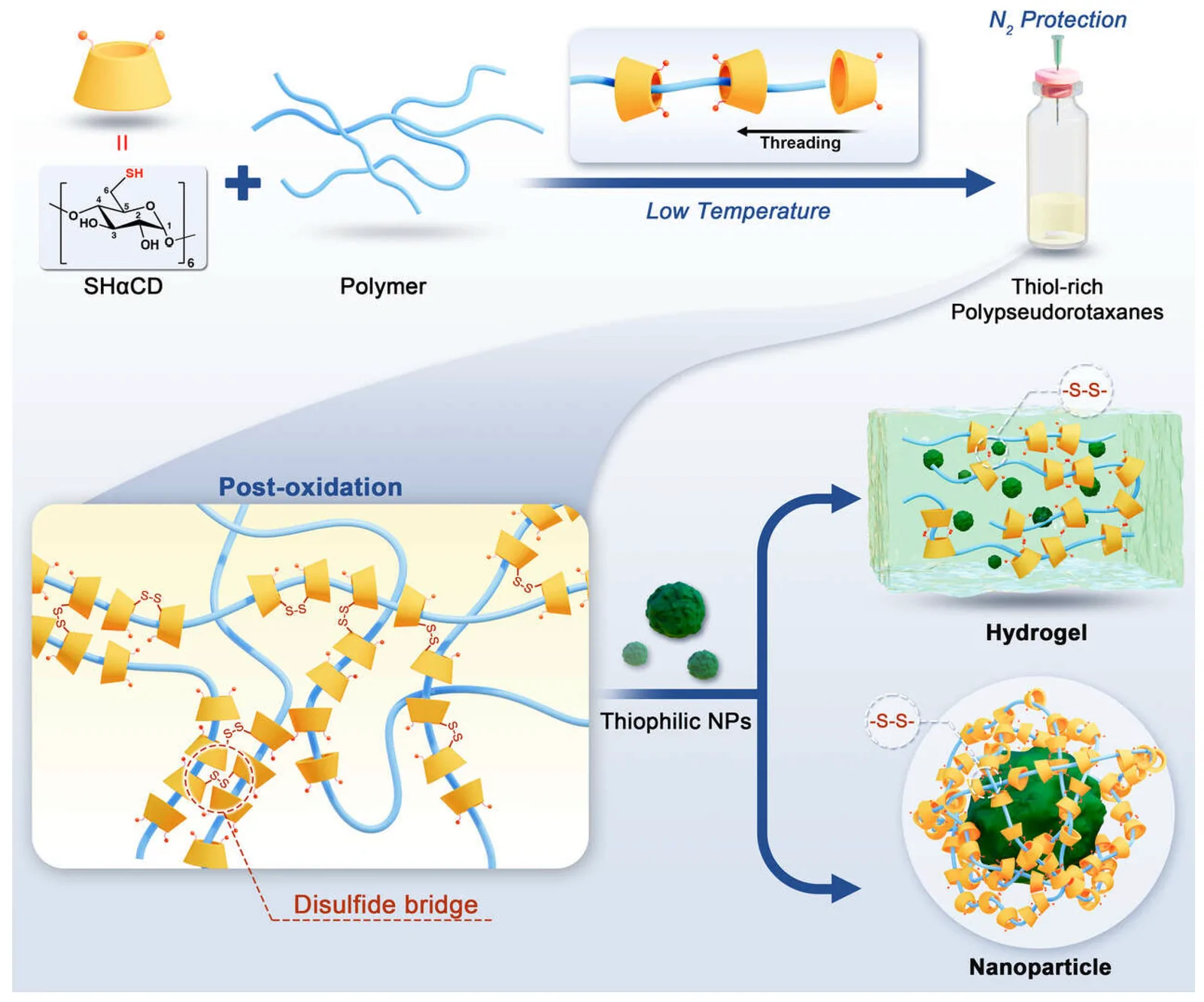
Schematic illustration of the one-pot formation of disulfide-crosslinked SPPRs by threading thiolated α-CDs onto polymer chains, followed by functionalization with thiophilic NPs to form reinforced hydrogel hybrids or SPPR-wrapped NPs. Reproduced from Ref. [72] under the terms of the Creative Commons Attribution 4.0 International License (CC BY 4.0). Copyright © 2025 The Authors.

Photothermal conversion can first assist hydrogel formation and mechanical reinforcement. As shown in Figure 6, Xu et al. prepared self-assembled thiol-rich polypseudorotaxanes (SPPRs) by threading thiolated α-CDs onto polymer chains and oxidizing the thiols to form disulfide-crosslinked hydrogels [72]. Thiophilic CuS and gold nanoparticles (AuNPs) were tethered to the network through sulfur–metal interfacial interactions, which reinforced the hydrogel. Under NIR irradiation, CuS-mediated heating accelerated thiol oxidation and disulfide-bond formation, enabling photothermally induced gelation within 15 min. At 37 °C, the $G^{\prime }$ values of the nanocomposite hydrogels increased from approximately 10 to 1000 Pa within 30 min. During alternating 1% and 120% strain tests, the hydrogels reversibly underwent gel–sol–gel transitions and recovered their elastic behaviour after the strain returned to 1%. At 37 °C, the dynamic disulfide crosslinks could be cleaved by 0.5 M DTT, resulting in a gel–sol transition within 12 h. In comparison, 0.1 M GSH progressively reduced the crosslinks and eroded the network. The resulting material combined NIR-triggered gelation with reduction-responsive disassembly and mechanical reinforcement.

Photothermal heating can also regulate reversible transitions in preformed networks. Kılıç and Ceylan used poly(vinylpyrrolidone) (PVP)-coated gold nanospheres (AuNS) and silver nanocubes (AgNCs) as thermoplasmonic components in physically crosslinked stearyl methacrylate/vinyl pyrrolidone hydrogels [14]. UV or visible-light irradiation increased the local temperature and melted crystalline domains formed by the long alkyl side chains. The associated decrease in modulus and increase in chain mobility enabled shape recovery and self-healing. The NP-containing hydrogels recovered their permanent shape within 1 min under the corresponding light stimulus and exhibited healing efficiencies of approximately 66–67% after heating at 37 °C for 3 min. After removal of the stimulus, recrystallization subsequently restored the fixed network state. Changing the particle type and concentration further altered the crystallinity, self-healing performance, and mechanical properties of the hydrogel. This provides a means of tuning the macroscopic properties of the hydrogel through NP composition. Photothermal nanomaterials can cooperate with thermal-responsive hydrogel to control drug release. In an LCST-type hydrogel, NIR-induced PNIPAM contraction generated an initial drug pulse followed by diffusion-limited sustained release [114]. At 37 °C, network contraction reached equilibrium within approximately 20 min. Each NIR cycle expelled approximately 30% of the hydrogel water, followed by recovery to nearly its initial mass after 3 days of cooling. This reversible response was maintained over the 21-day test period. In a CaCO_3_/PDA system, acidity initiated Ca^2+^-mediated gelation and ion release, whereas NIR heating accelerated CaCO_3_ decomposition and combined calcium overload with photothermal tumour ablation [115]. The photothermal response remained reproducible over three NIR on–off cycles.

Overall, photothermal nanomaterials connect optical stimulation with heat-dependent changes in hydrogel networks and therapeutic release. Through appropriate interfacial design, the same nanocomponent may assist gelation or reinforcement while regulating the release of drugs, gases, or ions. Photothermal conversion therefore provides a versatile route for coordinating remote activation with other chemical and thermal responses.

#### 2.3.2. Catalytic and Ultrasound-Responsive Nanomaterials

Catalytic nanomaterials convert endogenous substrates through Fenton or enzyme-mimetic reactions [24,56]. GOX-like activity generates gluconic acid, whereas peroxidase- and CAT-like activities convert $H_{2}O_{2}$ into ·OH or $O_{2}$ [17,24]. These reactions can remodel pathological microenvironments and generate chemical signals that cleave stimulus-labile bonds or promote therapeutic release [24,56].

Ultrasound complements endogenous catalysis by providing non-invasive and spatially controllable activation with relatively deep tissue penetration [116]. Acoustic energy can be transferred to hydrogels through thermal and non-thermal mechanisms [117]. Ultrasound-induced heating can accelerate molecular diffusion or induce network transitions, whereas cavitation and other mechanical effects can alter network permeability or cleave susceptible bonds [117]. Sonosensitizing nanomaterials additionally generate ROS under ultrasound irradiation, enabling remotely controlled sonodynamic therapy [15]. The resulting ROS may directly damage target cells or participate in subsequent network reactions [118]. When enzyme-mimetic and sonosensitizing functions coexist in one nanomaterial, continuous substrate conversion can be coupled with externally timed ROS production to amplify treatment [17].

Fenton catalysis can utilize endogenous $H_{2}O_{2}$ as a chemical trigger for prodrug activation. Huang et al. co-encapsulated TK-linked DOX prodrug micelles and 0.1 wt% ${\mathrm{Fe}}_{3}O_{4}$ NPs modified with PEG and branched polyethylenimine in a supramolecular hydrogel [56]. In the mildly acidic tumour environment, the NPs decomposed into ${\mathrm{Fe}}^{2+}$ and ${\mathrm{Fe}}^{3+}$, after which ${\mathrm{Fe}}^{2+}$ catalyzed the conversion of $H_{2}O_{2}$ into ·OH through a Fenton reaction. The resulting ·OH cleaved the TK linkages, causing micelle dissociation and DOX release. Methylene-blue degradation confirmed temperature-enhanced Fenton activity at pH 6.5 using 100 mM $H_{2}O_{2}$, although the assay did not provide absolute ·OH generation rates or catalytic kinetic parameters. Under 808 nm NIR irradiation, ${\mathrm{Fe}}_{3}O_{4}$-mediated heating raised the hydrogel temperature to approximately 41.9 °C within 5 min, thereby enhancing Fenton catalysis and accelerating drug release. Accordingly, cumulative DOX release over 72 h increased from 43.6% to 79.7% as the temperature increased from 37 to 45 °C. This design coupled acid- and $H_{2}O_{2}$-responsive prodrug activation with sustained chemotherapy and externally controlled photothermal enhancement [56].

**Figure 7:**
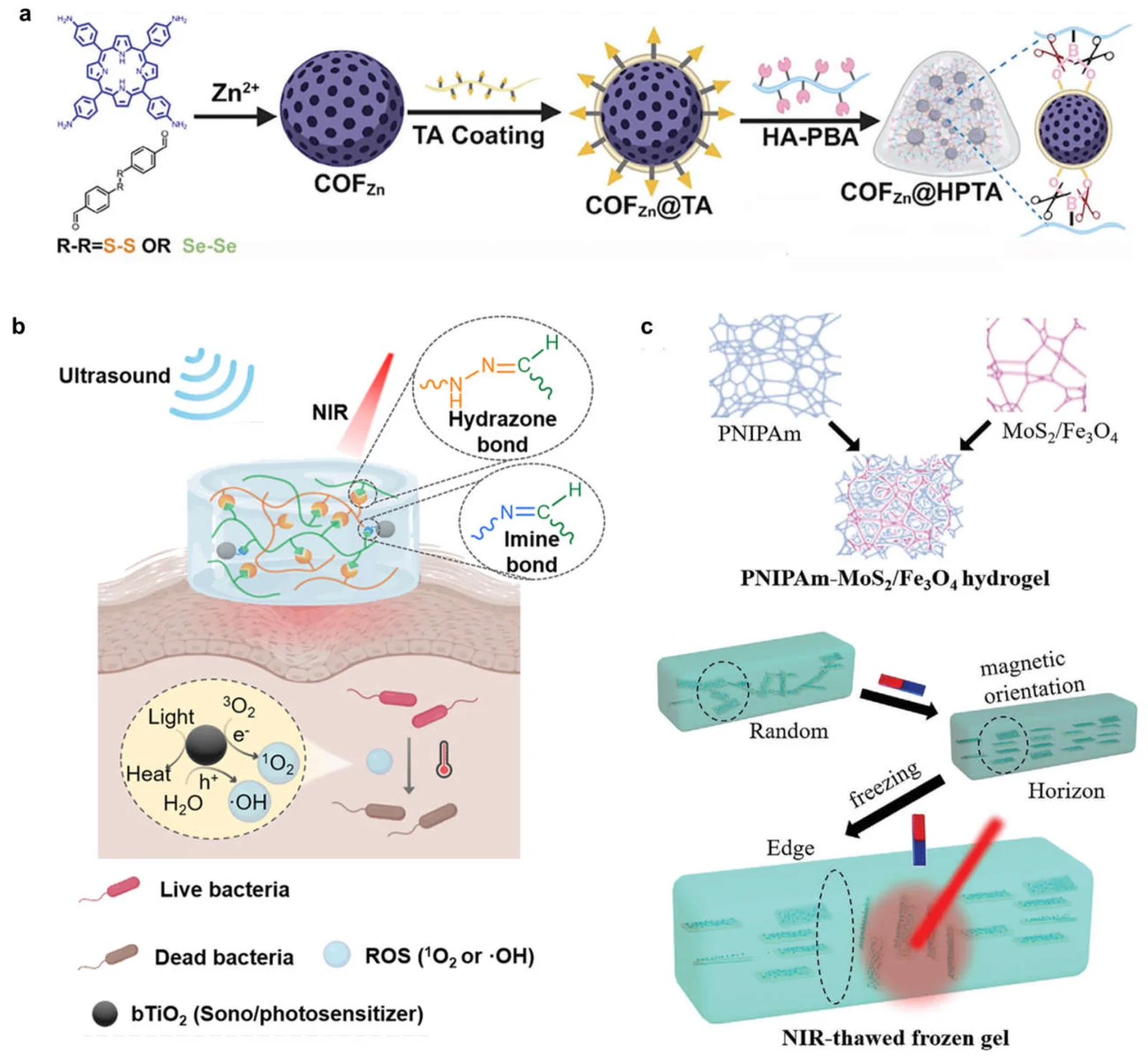
(**a**) Schematic preparation of a ROS-responsive and photothermally active hydrogel formed by connecting TA-coated Zn(II)-coordinated porphyrin COFs containing disulfide or diselenide linkages (COFZn@TA) with PBA-modified HA. Reproduced with permission from Ref. [24]. Copyright © 2025 Wiley-VCH GmbH. (**b**) An imine- and hydrazone-crosslinked network containing bTiO_2_ NPs that combines pH responsiveness with sonodynamic, photodynamic, and photothermal activities. Reproduced from Ref. [15] under the terms of the Creative Commons Attribution 4.0 International License (CC BY 4.0). Copyright © 2025 The Authors. (**c**) Schematic illustration of the fabrication of a PNIPAM–MoS_2_/Fe_3_O_4_ hydrogel, magnetic-field-induced orientation of MoS_2_/Fe_3_O_4_, and subsequent NIR treatment of the frozen hydrogel. Reproduced from Ref. [113] under the terms of the Creative Commons Attribution 4.0 International License (CC BY 4.0). Copyright © 2022 The Authors.

Enzyme-mimetic catalysis provides another route for using pathological metabolites as responsive inputs. Wu et al. synthesized Zn(II)-coordinated porphyrin covalent organic frameworks (COFs) containing disulfide or diselenide linkages and coated them with TA (Figure 7a) [24]. The resulting COFZn@TA NPs were connected to PBA-modified HA through dynamic boronate ester bonds to form an injectable adhesive hydrogel. These NP–polymer junctions increased the modulus and resistance to deformation. The incorporation of COF materials increased both rheological moduli, with $G^{\prime }$ remaining higher than $G^{\prime \prime }$ over 80% of the tested strain range. In addition, the COF materials exhibited GOX-like activity and lowered glucose concentrations in diabetic serum in vitro. For in vivo wound treatment, the hydrogel containing 400 μg/mL COFZn reduced local glucose levels within 12 h and maintained them at low levels for at least 24 h. However, the resulting glucose-conversion products and catalytic kinetic parameters were not reported. The disulfide or diselenide units within the NPs could be further cleaved by ROS to release Zn^2+^, providing antibacterial and tissue-repair functions. Under 808 nm NIR light irradiation, the wound-site temperature reached approximately 55 °C within 4 min, enabling photothermal antibacterial treatment. This multifunctional COF platform thus integrated glucose regulation, ROS-responsive ion release, network reinforcement, and photothermal activity.

Building on this functional integration, Xu et al. combined multiple enzyme-mimetic activities with ultrasound responsiveness in an injectable and self-healing hydrogel [17]. The hydrogel was formed from PDA-grafted sodium alginate, hydroxypropyl CS, and 2-formyl PBA through dynamic boronate ester and imine bonds. Gold-decorated titanium/iron (TF) bimetallic metal–organic framework (MOF) nanosheets (Au/TF-MOF) and DOX were incorporated into the network. The nanosheets functioned as both multifunctional nanozymes and sonosensitizers. Under acidic conditions, cleavage of the dynamic bonds promoted the release of DOX and the nanosheets. The Au/TF-MOF nanosheets exhibited both peroxidase-like and CAT-like activities, enabling the conversion of $H_{2}O_{2}$ into ·OH and $O_{2}$, respectively. Furthermore, their GOX-like activity consumed glucose and generated gluconic acid, enabling starvation therapy while simultaneously creating an acidic microenvironment that accelerated the pH-responsive hydrogel disassembly. The generated $O_{2}$ supplied the subsequent sonodynamic reaction, during which ultrasound-excited nanosheets produced singlet oxygen (O21). This catalytic cascade integrated starvation therapy, chemodynamic therapy, sonodynamic therapy, and chemotherapy, with ultrasound providing temporal control over ROS production [17]. Although probe-based O21 detection and time-dependent dissolved-oxygen measurements confirmed sonodynamic ROS generation and $H_{2}O_{2}$ decomposition, sonochemical yields and catalytic kinetic constants were not reported.

Surface functionalization can further transform ultrasound-responsive NPs from retained functional components into dynamic covalent network junctions while preserving their acoustic and optical activity [15]. As shown in Figure 7b, Wang et al. demonstrated this strategy by introducing amino-functionalized, mesoporous-silica-coated black ${\mathrm{TiO}}_{2}$ (bTiO_2_) NPs into a hydrazone-crosslinked network [15]. Surface amino groups on the NPs reacted with residual aldehydes on the polymer backbone to form interfacial imine bonds, thereby anchoring NPs as secondary crosslinking sites. Improved particle dispersion and additional crosslinking increased the compression modulus from approximately 60 to 83 kPa. After physical cutting and rejoining, the hydrogel recovered approximately 97% of its compression modulus within 24 h, while cyclic strain measurements showed rapid recovery of the storage modulus after high-strain deformation. The hydrazone and imine bonds also retained acid-dependent network dynamics. Under ultrasound irradiation at 1 MHz with a 50% duty cycle, b${\mathrm{TiO}}_{2}$ generated O21 and ·OH for sonodynamic antibacterial therapy. The associated cavitation additionally caused mechanical and thermal damage to bacterial membranes. Under NIR irradiation, the NPs generated ROS and heat for photodynamic and photothermal treatment and maintained a stable photothermal response over four heating–cooling cycles. Probe responses confirmed the generation of O21 and ·OH under ultrasound and NIR irradiation without providing absolute ROS-generation rates or sonochemical yields. The network therefore combined pH responsiveness with sonodynamic therapy, photodynamic therapy, and photothermal therapy [15].

Ultrasound may be used to initiate new crosslinks between nanomaterials and polymer chains after primary gelation. In this role, it can activate both nanomaterial-mediated therapy and secondary hydrogel crosslinking, extending its role from a therapeutic stimulus to an external signal for on-demand network regulation. Yan et al. demonstrated this strategy in a dextran/CS hydrogel combining pH-responsive imine bonds with ultrasound-responsive thiolated mesoporous silica-coated titanium dioxide NPs (${\mathrm{TiO}}_{2}$@MS-SH) [118]. Ultrasound at 120 kHz promoted thiol–norbornene reactions between the particle surfaces and polymer chains, immobilizing the NPs as multivalent crosslinkers. Following 30 min of sonication, the compressive modulus increased from 6.46±0.93 to 15.60±3.01 kPa, although longer exposure eventually caused cavitation-induced chain cleavage. The NPs simultaneously generated O21 for sonodynamic antibacterial therapy. Probe-based measurements indicated maximal O21 generation at 3 wt% ${\mathrm{TiO}}_{2}$@MS-SH, whereas increasing the loading to 5 wt% reduced ROS generation as a result of particle aggregation. Data on absolute O21-generation rates and sonochemical yields, however, were not reported. Under acidic conditions, imine-bond hydrolysis loosened the network and increased CS protonation, strengthening contact-based antibacterial activity and improving ROS access to bacteria. Ultrasound thus served as both a crosslinking signal and a therapeutic trigger, while ${\mathrm{TiO}}_{2}$@MS-SH acted as both a reactive crosslinker and a sonosensitizer [118].

In summary, these nanomaterials contribute to hydrogel design at multiple levels, from catalytic regulation of pathological microenvironments and cargo activation to ultrasound-triggered therapy. Surface engineering further allows ultrasound-responsive nanomaterials to reinforce preformed networks or participate in secondary crosslinking. The convergence of catalytic activity and sonosensitization within single nanomaterials further enables synergistic cascade reactions and multi-functional therapy [17,24]. However, catalytic activity and ultrasound-generated ROS must be precisely controlled to achieve adequate therapeutic efficacy without causing excessive oxidative damage.

#### 2.3.3. Magnetically Responsive Nanomaterials

Magnetic nanoparticles (MNPs) are distinguished by their ability to translate magnetic fields into particle reorganization or localized heating within hydrogel networks [119]. Static or rotating magnetic fields induce field-directed particle distribution and orientation, whereas alternating magnetic fields generate magnetothermal effects. These responses can regulate transport pathways, establish anisotropic structures, or trigger thermoresponsive network transitions [120]. Through suitable surface and network design, magnetic nanomaterials may therefore regulate both hydrogel architecture and multi-stimuli behaviour.

MNPs can couple field-regulated drug transport with the intrinsic responsiveness of a hydrogel [119]. In an imine-crosslinked dextran hydrogel containing Fe_3_O_4_ NPs, acidic conditions promoted network swelling and drug release, whereas a static magnetic field strengthened interparticle association and suppressed transport [121]. The two stimuli therefore regulated release through complementary changes in network connectivity and effective diffusion pathways. When MNPs are retained mainly through physical or noncovalent interactions, aggregation or diffusion may limit their contribution to permanent network organization [119]. Surface functionalization can instead immobilize them as network junctions and orientation-directing units, allowing magnetic fields to regulate both hydrogel mechanics and structural anisotropy [23]. Weng et al. introduced carboxyl-functionalized MNPs into methacrylated collagen peptide (COPMA) hydrogels [23]. Hydrogen bonding between the particles and polymer chains allowed the MNPs to act as physical junctions and increased the storage modulus by approximately 3.2-fold. The hydrogels also recovered their initial mechanical properties during alternating 1% and 500% strain cycles, providing evidence of short-term mechanical durability under repeated deformation. The authors then modified the particle surfaces with dopamine methacrylamide (DMA) to obtain MNP@DMA. Under UV irradiation, the methacrylamide groups were covalently incorporated into COPMA, while hydrogen bonding and surface coordination retained reversible associations. This combined covalent and physical crosslinking improved particle fixation and preserved self-healing capacity [23]. A magnetic field aligned the particles into chain-like structures during gelation, and subsequent UV crosslinking fixed the anisotropy. The injectable precursor could therefore form anisotropic hydrogels within irregular geometries. The aligned NPs guided the spreading of human bone marrow-derived mesenchymal stem cells and influenced their ligament- or tendon-related differentiation. In this system, the magnetic field directed structural organization, whereas light fixed the network. The nanomaterials therefore functioned as both crosslinking sites and morphology-guiding units [23].

Magnetically controlled orientation can also regulate photothermal and thermoresponsive behaviour [120]. As shown in Figure 7c, Chen et al. anchored ${\mathrm{Fe}}_{3}O_{4}$ NPs and thermosensitive PNIPAM onto molybdenum disulfide (${\mathrm{MoS}}_{2}$) nanosheets to construct magnetic- and NIR-responsive hydrogels [113]. Surface coordination and other noncovalent interactions drove the composite nanosheets to assemble into fibrillar structures. The superparamagnetism provided by Fe_3_O_4_ enabled rapid alignment either parallel or perpendicular to the incident NIR light. Under 808 nm NIR irradiation for 2 min, the perpendicular and parallel configurations reached 55.2 and 44.1 °C, respectively, demonstrating the dependence of photothermal conversion on nanosheet orientation. In a 5 mm thick hydrogel, the resulting PNIPAM response occurred within 240 s and triggered the release of fluorescent molecules, with faster release in the perpendicular configuration. The nanomaterials thus acted as both self-assembling structural units and magnetic–photothermal response elements. Magnetic programming and localized irradiation further enabled blockwise control of hydrogel anisotropy for data storage and molecular release [113]. Beyond using magnetic alignment to regulate photothermal efficiency, optical and magnetic heating can provide complementary external inputs for thermally controlled release [119]. Yang et al. coated ${\mathrm{Fe}}_{3}O_{4}$ nanoclusters with silica and then wrapped them with amino-functionalized ${\mathrm{Ti}}_{3}C_{2}T_{x}$ MXene to obtain MXene-wrapped MNPs (MNPs@MXene) [18]. The particles were incorporated into a dual-network hydrogel composed of covalently crosslinked PNIPAM and ionically crosslinked alginate. NIR irradiation and an alternating magnetic field (AMF) both remotely heated the hydrogel and induced PNIPAM contraction. The heating response could be repeatedly switched on and off over five NIR or AMF cycles, enabling repeatable release of loaded AgNPs. NIR-triggered treatment promoted the repair of epidermal and subcutaneous infected wounds in diabetic rats. Magnetic activation was evaluated only in vitro, however, so its proposed advantage for deeper wounds requires in vivo validation [18].

MNPs regulate hydrogels through field-directed structural reorganization and magnetothermal energy conversion. Their functions extend from controlling molecular transport to reinforcing networks, fixing anisotropic architectures, and triggering release. Coupling these effects with pH, thermal, or light responses broadens the available modes of multi-stimuli regulation. Their effective use depends on stable particle retention and quantifiable coupling between the applied field and the resulting network response. These design principles are subsequently related to the coordination and functional roles of different responses in biomedical applications.

Functional nanomaterials allow hydrogels to couple multiple endogenous and externally applied stimuli with network regulation and therapeutic activity. This functional integration is advantageous for achieving localized and temporally controlled responses, but it also introduces composition- and dose-dependent safety concerns. The loading fraction of nanomaterials should be specified because it affects energy conversion, network behaviour, and biological safety. Detailed activation conditions are required to judge whether the intended response can be safely and reproducibly achieved within physiologically relevant environments and tissue depths. Quantitative transduction measurements are required to relate stimulus exposure to material response and thereby define effective and safe operating windows. This is particularly important for sonosensitizers and catalytic nanomaterials because insufficiently controlled reactive species may damage surrounding tissues. Biocompatibility, retention, and clearance should also be evaluated to assess local persistence and systemic safety. As summarized in Table 3, thermal responses and short-term cytocompatibility were frequently reported, whereas catalytic kinetics, ROS generation, sonochemical yields, magnetic-field conditions, and long-term nanomaterial fate and safety were rarely examined. The table consolidates the available quantitative evidence, while recurring evidence gaps are clarified in the accompanying table note rather than repeated in individual entries.

## 3. Biomedical Applications

Multi-stimuli-responsive hydrogels have been investigated in biomedical environments where biochemical cues and treatment requirements vary across anatomical locations and over time [7,122]. In wound treatment and in vivo drug delivery, complementary endogenous signals can support pathology-dependent network changes and therapeutic release [29,122], while external inputs such as light, ultrasound, or magnetic fields provide additional on-demand control [20,123]. In tissue engineering, multiple stimuli can also regulate scaffold structure, mechanical properties, and fabrication processes, thereby supporting cell organization and tissue regeneration [23,87]. The following sections examine these application-specific functions in wound treatment, in vivo drug delivery and cancer therapy, and tissue engineering and regeneration.

### 3.1. Wound Treatment

Wound healing proceeds through overlapping inflammatory, proliferative, and remodeling phases, during which the local biochemical and physical conditions continuously change [3,12]. Infection and chronic inflammation can alter the pH of a wound. They may also increase the activity of proteolytic enzymes and the accumulation of ROS [1,3,124]. Chronic hyperglycemia further impairs endothelial function and angiogenesis in diabetic wounds [3,123]. In addition, the levels and spatial distributions of these signals may vary among wound types and healing stages. Complementary wound signals can improve the distinction between infection, oxidative damage, and metabolic dysfunction and assign the resulting hydrogel responses to different therapeutic tasks [12,103].

Representative designs combine pH responsiveness with sensitivity to enzymes, ROS, or glucose because these signals reflect different aspects of infection and tissue damage [12,103,124]. For example, pH- and enzyme-responsive hydrogels can combine changes caused by bacterial metabolism with the increased activity of bacterial or tissue-derived enzymes, thereby reducing dependence on nonspecific pH variation alone [53,66,124]. Similarly, pH- and ROS-responsive networks can undergo accelerated degradation and therapeutic release under acidic and oxidative conditions associated with infected or inflamed wounds [103,125]. In diabetic wounds, glucose and pH provide complementary metabolic and chemical signals [126,127]. Li et al. developed a glucose- and pH-responsive hydrogel that released an antimicrobial peptide and pravastatin-loaded nanomaterials while also scavenging ROS [127]. After 14 days, 70% and 62% of the hydrogel mass remained at glucose concentrations of 10 and 40 mM, respectively, whereas only 40% remained at pH 5.0. After 5 days, pravastatin and antimicrobial peptide release increased from 50.2% and 64.4% at pH 7.4 to 64.5% and 73.6% in the presence of 40 mM glucose, and to 80.8% and 83.7% at pH 5.0, confirming that both pathological stimuli accelerated therapeutic release. The resulting hydrogel reduced bacterial infection and inflammatory cytokine levels, thereby promoting angiogenesis in methicillin-resistant *Staphylococcus aureus*-infected diabetic wounds. This example illustrates that multi-responsive hydrogels can respond to different pathological signals to regulate drug delivery while simultaneously addressing infection, oxidative stress, and vascular dysfunction. However, the pH 5.0 condition used to maximize degradation and release is lower than the pH measured in many chronic wounds. Therefore, the contribution of acid-triggered release to the observed therapeutic effect in vivo remains unresolved. ROS responsiveness should nevertheless be distinguished from ROS-scavenging capacity. ROS responsiveness refers to ROS-induced network changes or cargo release, whereas ROS scavenging depends on the antioxidant activity of the hydrogel components or the incorporated therapeutic agents [103,125]. Combining these functions allows ROS to act as both a pathological trigger and a therapeutic target [22,125]. Beyond this dual role of ROS, glucose-responsive systems can consume glucose and combine metabolic regulation with antibacterial treatment [12,24]. CAT-mediated oxygen generation can further relieve hypoxia, while the released therapeutic agents regulate inflammation and promote angiogenesis [12]. These combined functions allow the hydrogel to connect the recognition of infection, oxidative stress, and metabolic dysfunction with antibacterial treatment and subsequent tissue repair. However, the process of GOX-mediated glucose oxidation consumes O_2_ and generates H_2_O_2_. It remains unclear whether CAT-mediated O_2_ regeneration sufficiently compensates for this oxygen depletion. The balance between antibacterial ROS generation and H_2_O_2_-induced oxidative stress also requires quantitative evaluation to ensure antibacterial efficacy without injuring host tissue. Externally applied NIR or alternating magnetic fields can provide complementary and temporal controlled over wound treatment [18,123]. In one wound-monitoring system, NIR-triggered nitric oxide (NO) release provided antibacterial and anti-inflammatory effects, while temperature responsiveness enabled simultaneous wound monitoring [123]. In another system, NIR and magnetic activation enabled repeatable AgNP release, with NIR treatment promoting infected diabetic wound repair in vivo, whereas magnetic control was evaluated only in vitro [18].

In addition to recognizing coexisting pathological signals, multi-stimuli-responsive hydrogels can match therapeutic functions to the changing requirements of wound healing. During the early inflammatory stage, bacterial infection, excessive ROS, and persistent inflammation need to be controlled [12,103]. As the wound enters the proliferative and remodeling stages, treatment priorities shift toward macrophage regulation, angiogenesis, collagen deposition, and tissue regeneration [12,125]. Loading therapeutic agents at different positions within a hydrogel can produce rapid and sustained release profiles that broadly correspond to different wound-healing requirements [128]. When these profiles arise from spatial loading and sequential release barriers, the system provides programmed spatiotemporal delivery rather than direct identification of the healing stage [128]. Wu et al. demonstrated this strategy using a pH- and ROS-responsive hydrogel containing diclofenac sodium (DS) and mangiferin (MF) [128]. In this system, DS was directly embedded in the hydrogel, whereas MF was first encapsulated in the core of pH-responsive micelles. Under acidic and oxidative conditions, the hydrogel disintegrated within 6 h at pH 5.0 or with 1 mM H_2_O_2_, enabling rapid DS release to inhibit early inflammation. When both stimuli were applied together, DS release reached 84.5% at 24 h. By contrast, the release of MF required the sequential collapse of the hydrogel and micelles. It increased from 22.6% at pH 7.4 to 36.9% at pH 5.0 within 24 h and was followed by sustained release for over 7 days. These distinct release profiles were proposed to provide rapid early anti-inflammatory treatment followed by more sustained regenerative support. The pH- and ROS-responsive mechanisms enabled inflammation-dependent release, whereas the spatial separation of the two agents and the additional micellar barrier were essential for producing the different release profiles. Although acidic and oxidative conditions accelerated the two release processes, the relative contribution of acid-triggered release to the observed profiles in vivo was not separately resolved.

Related systems have coordinated early infection control with subsequent regulation of the wound microenvironment. In a pH- and ROS-responsive glycopeptide hydrogel, the network disintegrated within 6 h at pH 5.0 or in the presence of 1 mM H_2_O_2_ [125]. The release of astragaloside IV at 24 h increased from 26.3% under the control condition to 59.1% under combined stimulation with pH 5.5 and 0.1 mM H_2_O_2_. The released agent subsequently reduced oxidative stress and inflammation, regulated macrophage polarization, and promoted angiogenesis. Because the network response was evaluated mainly at pH 5.0–5.5, the relevance of this strongly acidic activation window to the in vivo wound environment remains uncertain. Pei et al. further developed a bacteria-responsive hydrogel containing pH-sensitive nanozymes and pH/lipase-responsive micelles [124]. Changes in bacterial metabolites, pH, and lipase activity activated a cascade treatment combining chemodynamic therapy and self-driven NO release during the infection stage. After infection control, the multiple nanozyme activities relieved oxidative stress and promoted angiogenesis, thereby connecting self-activating antibacterial treatment with post-infection tissue regeneration [124]. However, the exposure thresholds separating therapeutically effective levels of reactive species and NO from potential damage to host cells or regenerating tissue were not established under representative wound conditions. Furthermore, although localized bacterial activity may generate acidic microenvironments, whether the tested pH 5.5–6.5 is sufficiently sustained within chronic wounds to activate the system in vivo remains uncertain. These examples show that phase-matched treatment may be achieved by multi-stimuli-responsive hydrogels through either different release barriers or a programmed change from antibacterial action to microenvironment regulation.

More advanced systems attempt to adjust therapeutic output according to changes in the wound state rather than relying only on a predetermined release program. Fu et al. constructed a pH- and $H_{2}O_{2}$-responsive hydrogel containing Cu-doped oxygen-vacancy ${\mathrm{MoO}}_{3-x}$ nanozymes and curcumin [129]. Alkaline conditions and elevated H_2_O_2_ independently accelerated the release of the nanozymes and curcumin. At 24 h, nanozyme release increased from 45.6% at pH 7.4 to 62.5% at pH 8.5 and to 72.3% in the presence of 10 mM H_2_O_2_ at pH 7.4, while curcumin release increased from 42.1% to 58.3% and 68.9% under the corresponding conditions. The released nanozymes catalyzed the decomposition of $H_{2}O_{2}$ into oxygen, thereby reducing oxidative stress and alleviating wound hypoxia. Curcumin inhibited bacterial growth and reduced the viability of mature biofilms. Time-dependent measurements showed that initially high pH and ROS levels promoted therapeutic release, whereas treatment reduced these pathological signals and slowed further release. The authors therefore described the system as a closed-loop therapeutic platform in which pathological signals regulated release and the resulting treatment, in turn, reduced those signals [129]. The in vivo fate of the metal-containing nanozymes was also examined. Quantitative analysis showed that Mo and Cu were initially enriched in the wound tissue and kidneys, but their tissue concentrations progressively decreased over 28 days, accompanied by urinary excretion and no evidence of persistent accumulation in major organs. This design represents a step beyond fixed sequential delivery by linking treatment intensity to the changing wound microenvironment.

Overall, multi-stimuli-responsive hydrogels can coordinate infection control, oxidative-stress regulation, and regenerative support within the changing wound microenvironment. However, response conditions established in laboratory buffers may not translate directly to clinical wounds, where pH and other pathological signals vary with wound type, infection status, and healing stage [3]. In particular, although many chronic wounds exhibit neutral-to-alkaline bulk pH, several hydrogels characterized under acidic conditions have nevertheless achieved substantial wound-healing effects in vivo [124,125,127,128]. These therapeutic outcomes do not independently establish acid-triggered activation within the wound because other stimuli, pH-independent release, and spatial or temporal pH heterogeneity may also contribute. Further studies are therefore required to determine how spatial and temporal pH variations within wounds affect hydrogel activation and to what extent pH-responsive activation contributes to therapeutic performance in vivo [4,128]. Beyond pH, exudate composition, ionic strength, mechanical movement, and bacterial biofilms may further affect hydrogel stability, adhesion, and therapeutic release [3,130]. As wound-healing stages overlap and progress at different rates, predetermined sequential release profiles may not remain synchronized with the actual healing process. Future studies should evaluate hydrogel responses in representative wound fluids and chronic wound models and determine whether infection control and regenerative delivery remain appropriately coordinated during healing. To provide a structured comparison of the current evidence, Table 4 summarizes the experimentally applied stimulus ranges, quantitative material and release responses, pathological relevance, therapeutic outcomes, validation levels, and reported biosafety evidence of the representative wound-treatment systems discussed above.

### 3.2. In Vivo Drug Delivery

In vivo drug delivery faces more complex physiological barriers and more pronounced lesion heterogeneity [7]. Along the gastrointestinal tract, variations in pH, redox conditions, digestive enzymes, and microbial metabolism provide sequential signals for site-specific drug release [9,10]. In diabetes, fluctuations in glucose concentration constitute the principal disease-associated signal, while local pH may further influence hydrogel permeability and insulin release [8,51]. Inflamed joints are commonly associated with local acidification and elevated ROS [36,131], whereas tumour tissues contain multiple pathological signals distributed across extracellular and intracellular, as well as regional tissue environments [7,132]. These differences provide a basis for designing multi-responsive hydrogels that adapt drug retention and release to the administration route, disease state, or spatial organization of pathological signals.

In non-tumour diseases, multi-responsive designs are primarily employed to adapt to changes along the administration route and to establish disease-state-dependent release. Changes in pH, redox potential, and microbial enzyme activity along the gastrointestinal tract can be assigned to different steps of cargo protection and release. A pH- and redox-responsive hydrogel used the transition from gastric to intestinal conditions to retain Mg^2+^ during gastric passage and subsequently promote its release upon entering the reducing intestinal environment [10]. The release of Mg^2+^ reached 20.4% at pH 1.2 within 6 h, compared with 72.5% under combined pH 6.8 and reducing conditions. Similarly, a pH- and enzyme-responsive system restricted metronidazole release in the upper gastrointestinal tract, whereas azoreductase produced by anaerobic colonic bacteria accelerated its release at the target site [9]. Metronidazole release was minimal at pH 1.2 and was significantly enhanced under the cecal condition at pH 6.5. These systems demonstrate how multiple physiological signals along an administration route can be translated into sequential cargo protection and site-specific release.

**Figure 8:**
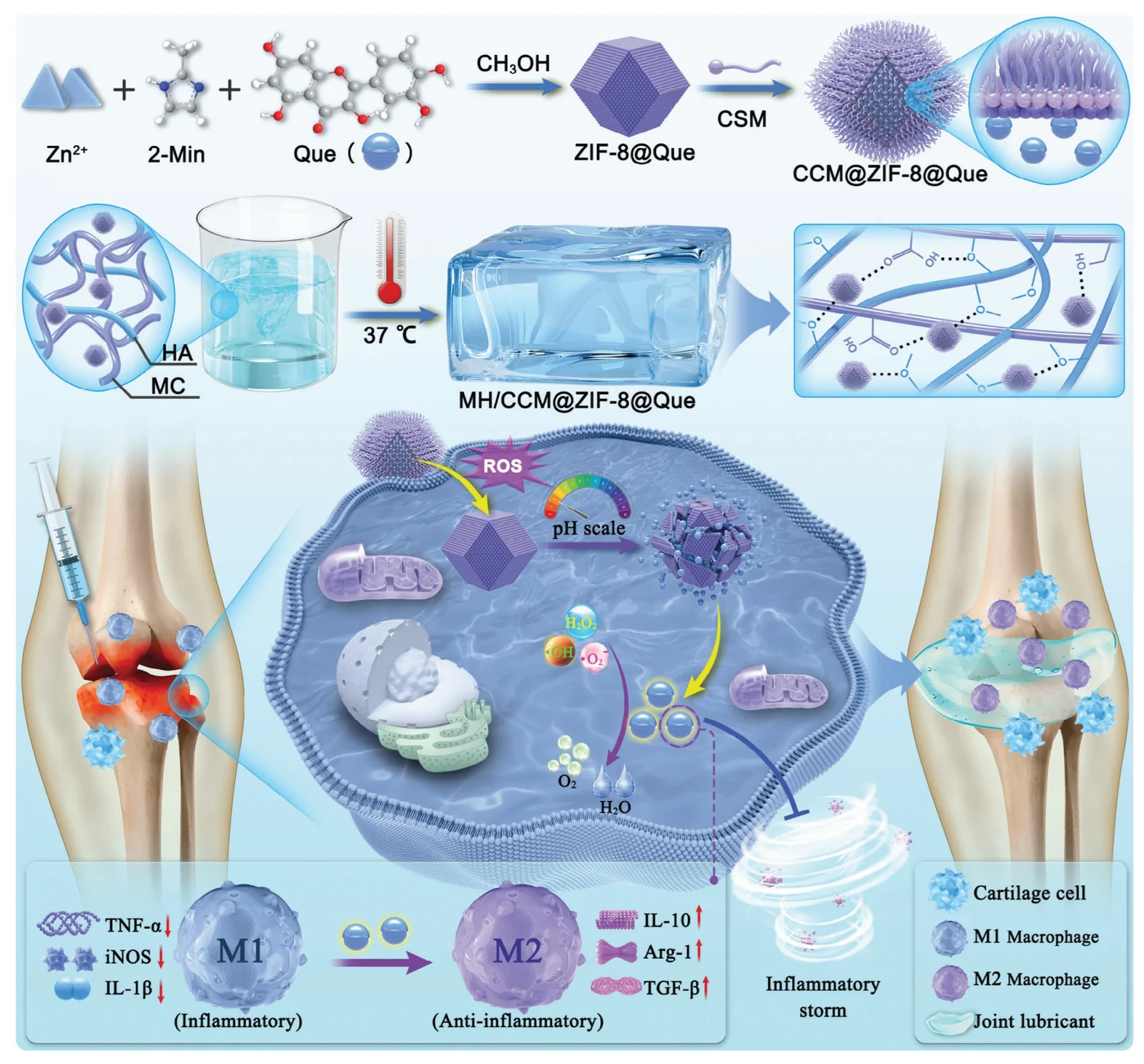
Schematic illustration of the preparation of quercetin-loaded NPs and their incorporation into a thermosensitive hydrogel that undergoes gelation at body temperature. The acidic and ROS-rich inflammatory microenvironment promotes the release of quercetin and Zn^2+^, thereby reducing oxidative stress and inflammation, promoting macrophage polarization toward the M2 phenotype, and protecting cartilage against osteoarthritic degradation. Reproduced with permission from Ref. [36]. Copyright © 2024 Wiley-VCH GmbH.

Arthritis is characterized by persistent inflammation, local acidification, oxidative stress, and progressive damage to synovial and cartilage tissues [131,133]. Meanwhile, rapid clearance of drugs from the joint cavity can shorten their therapeutic duration and necessitate repeated intra-articular administration [36,131]. Multi-responsive hydrogels can function as local drug reservoirs while using inflammation-associated pH and ROS changes to regulate therapeutic release and remodel the joint microenvironment. Zhang et al. developed a thermosensitive hydrogel that remained injectable at low temperature and underwent a sol–gel transition near body temperature, thereby providing joint lubrication and a local drug reservoir (Figure 8) [36]. The acidic and ROS-rich inflammatory environment accelerated the release of quercetin and ${\mathrm{Zn}}^{2+}$, thereby reducing oxidative stress, synovial inflammation, and cartilage degradation [36]. Although Zn^2+^ release and concentration-dependent cytocompatibility were evaluated in this study, evidence regarding the intra-articular retention, tissue distribution, and clearance of the Zn-containing NPs remains lacking. Related pH- and ROS-responsive systems provided sustained peptide release or inflammation-dependent methotrexate delivery, supporting chondrocyte activity and local anti-inflammatory treatment [131,134]. Across these systems, drug release was slower under physiological joint conditions and accelerated under acidic or ROS-rich conditions associated with joint inflammation [36,131,134]. Their prolonged intra-articular release may reduce the need for repeated administration, although the effects on injection frequency and injection-related complications require further in vivo evaluation.

For diabetes treatment, multi-responsive hydrogels can couple glucose-regulated insulin release with pH-dependent changes in network permeability or stability. In these systems, glucose serves as the principal disease-associated signal, whereas pH regulates the network state and consequently facilitates or restricts insulin diffusion. For example, a pH- and glucose-responsive hydrogel was constructed by Ali et al., in which glucose- and acid-induced network changes accelerated insulin release [8]. Under 100 mM glucose, insulin release at 24 h reached approximately 97% at pH 4.0 but only 51% and 48% at pH 7.5 and 10.0, respectively. Following subcutaneous administration in diabetic rats, the hydrogel produced a longer glucose-lowering effect than free insulin. However, the strongly acidic conditions and the glucose concentrations of 100–500 mM tested in vitro do not represent the typical subcutaneous diabetic environment. In another system, exposure to pH 4.5 induced network contraction and restricted insulin diffusion, whereas the hydrated network permitted glucose-regulated pulsatile release near physiological pH [51]. At pH 7.4, glucose-regulated insulin release was evaluated at glucose concentrations of 1 and 4 g/L, with the glucose-free condition used as the control. In this study, pH 7.4 approximated the subcutaneous environment, whereas pH 4.5 was used primarily to demonstrate acid-induced network contraction. These comparisons indicate that the relevance of glucose-responsive insulin delivery should be evaluated by considering the glucose-response range together with network stability and release performance near physiological pH.

Tumour-associated pathological signals are spatially distributed and may differ across tumour regions and cellular environments [7,132]. The extracellular tumour environment is commonly mildly acidic, whereas intracellular GSH concentrations are substantially higher than those in extracellular fluids [7,132]. The concentrations of ROS, enzyme activity, and the extent of hypoxia may also vary among tumour regions and change during disease progression and treatment [7,21,64]. Multi-responsive systems therefore do not require all signals to act simultaneously at the same location. Instead, different stimuli should be assigned to the sequential regulation of local retention, network degradation, and intracellular drug release. The design of pH- and GSH-responsive systems can exploit the extracellular tumour acidity to initiate carrier changes and the intracellular reducing environment to promote subsequent drug release [11,58]. Multi-responsive hydrogels are also particularly suited to postoperative tumour management. Their injectability allows them to fill irregular resection cavities and retain therapeutic agents around residual cancer cells [21,135]. Body temperature can first induce in situ gelation, after which local acidity, ROS, or intracellular reducing conditions regulate drug release. As demonstrated by a temperature- and pH-responsive system that serves as an injectable in situ delivery platform for local brain tumour therapy, the hydrogel supported sustained paclitaxel release in physiological environments (pH 7.4), and the retention of the drug was further prolonged in tumour environments (pH 6.5) [37]. This illustrates its potential to reduce dosing frequency and prevent glioblastoma recurrence.

More complex postoperative platforms can coordinate local drug retention with chemotherapy, immunotherapy, or ferroptosis. Li et al. developed a ROS-responsive thermosensitive polypeptide hydrogel that released DOX, resiquimod, and an antibody as the hydrogel degraded in response to ROS. The combined treatment of local chemotherapy with systemic antitumour immunity effectively inhibited postoperative tumour recurrence by inducing immunogenic cell death and eliciting long-term immune memory [21]. In another system, the hydrogel gelled within 30 s, supporting rapid fixation and local retention after administration [135]. At pH 6.5, around 65% of the supramolecular prodrug and 86% of the antibody were released by day 10, after which intracellular redox conditions activated the released prodrug and induced ferroptosis. The combination with immune checkpoint blockade further facilitated the elimination of residual tumour cells. Multi-responsive designs can therefore integrate resection-cavity filling, prolonged local retention, pathology-triggered release, and recurrence suppression within a single platform. Such injectable platforms prolong drug retention at the surgical site while minimizing systemic exposure during the critical phase of postoperative tissue repair. Furthermore, multi-responsive systems can convert tumour metabolic reactions into amplified signals that drive therapeutic cascades. Ding et al. incorporated GOX and the hypoxia-activated drug tirapazamine into a pH-, $H_{2}O_{2}$-, and GSH-responsive diselenide-crosslinked hydrogel [64]. Glucose oxidation by GOX generated gluconic acid and $H_{2}O_{2}$, which accelerated hydrogel degradation and drug release. The released selenium-containing products promoted GSH consumption. Concurrent glucose and oxygen depletion increased hypoxia and activated hypoxia-sensitive drug tirapazamine, thereby linking metabolic regulation, redox disruption, and hypoxia-activated chemotherapy [64]. Nevertheless, the extent of ROS generation and GSH depletion, as well as their potential effects on the redox homeostasis of surrounding healthy tissues, requires further evaluation.

The incorporation of exogenous physical stimuli together with endogenous pathological signals provides additional control over the onset, duration, and intensity of treatment. In addition to regulating drug release, externally delivered energy can be converted into heat or reactive species to initiate photothermal, photodynamic, sonodynamic, or chemodynamic treatment. In these systems, NIR light is commonly converted into heat by incorporating photothermal components into hydrogels [115,136]. The resulting local temperature increase not only enables photothermal therapy but also increases cell-membrane permeability. This can facilitate the cellular uptake of chemotherapeutics, thereby strengthening the synergistic effect of photothermal-chemotherapy [16,137]. In one representative system, NIR-triggered quercetin release inhibited heat-shock protein 70 and reduced tumour thermotolerance, enabling effective ablation at lower nanocage concentrations and irradiation intensities [138]. In another thermolabile-linker system, photothermal heating can induce the shrinkage of thermoresponsive networks, allowing repeatable pulsatile release upon NIR irradiation [114]. This design enables prolonged drug release with reduced frequency of drug administration, which is particularly valuable for tumours requiring long-term local therapy. Ultrasound has similarly coordinated pH-triggered component release with catalytic and sonodynamic therapy, as discussed in Section 2.3 [17]. Although short-term in vivo evaluation showed stable body weight and normal major-organ histology in this study, local oxidative injury to surrounding healthy tissue and the safe exposure range for sonodynamic treatment were not specifically evaluated. Magnetic fields provide an additional external means of regulating drug release. For example, a pH- and magnetic-responsive dextran hydrogel exhibited acid-accelerated and static-field-suppressed release of doxorubicin hydrochloride [121]. These systems illustrate how external and pathological signals can be linked through sequential or mutually reinforcing pathways rather than acting as independent switches.

Overall, multi-stimuli-responsive delivery systems can coordinate cargo protection, local retention, and release across different administration routes, disease states, and tissue compartments. The combination of endogenous pathological signals with externally applied energy further enables on-demand release and multimodal cancer treatment. Nevertheless, heterogeneous in vivo conditions and uneven energy deposition may alter response thresholds, reduce activation in deeper tissues, or damage surrounding healthy tissues. Magnetic-field-regulated release, in particular, remains largely validated in vitro [121]. Future studies should quantify tissue- and compartment-specific activation and establish the in vivo safety and therapeutic efficacy of remote triggering strategies. To provide a structured comparison of the current evidence, Table 5 summarizes the experimentally applied stimulus ranges, quantitative material and release responses, pathological relevance, therapeutic outcomes, validation levels, and reported biosafety evidence of representative multi-stimuli-responsive systems for in vivo drug delivery.

### 3.3. Tissue Engineering

In tissue engineering, the roles of multi-stimuli-responsive hydrogels extend beyond injectable matrices for local scaffold formation and bioactive-factor retention for the controlled release of therapeutic components. Their distinctive role lies in coordinating the adaptability of the scaffold with cell–matrix interactions, or in synergistically regulating the pathological environment to support tissue regeneration [23,139,140]. Their hydrated three-dimensional networks can support cell growth and matrix formation while facilitating nutrient transport and the presentation of regenerative signals [1,141,142]. Depending on the target tissue, an effective hydrogel scaffold should initially possess sufficient injectability and structural stability for reliable implantation and retention during the early stage of repair, while subsequently allowing cell infiltration, matrix remodeling, and integration with newly formed tissue [143]. Its mechanical evolution and degradation rates should therefore be coordinated with tissue formation rather than considered independently [1,143].

Studies have demonstrated that stimulus-triggered assembly and structural fixation can be used to construct scaffolds that direct cell behaviour. Li et al. used temperature changes, ion induction, and pH switching to regulate the self-assembly of biphenyl tripeptides into nanotubes or nanofibers [141]. These nanostructures subsequently formed a three-dimensional scaffold with a nanofibrous morphology resembling that of natural collagen fibers and supported cell spreading and proliferation in vitro. When rabbit chondrocytes were encapsulated and cultured for up to 21 days, increased cell proliferation and cartilage-associated matrix secretion were observed. The gene-expression and histological results were also consistent with maintenance of a hyaline cartilage-associated phenotype. However, these findings were limited to encapsulated cells cultured in vitro. Infiltration by surrounding cells, integration with native cartilage, and cartilage repair in vivo were not evaluated. Therefore, the translational relevance of this scaffold remains to be established.

Multiple stimuli can further regulate scaffold anisotropy and the mechanical environment perceived by cells. In the resulting anisotropic scaffold, magnetic alignment guided the spreading and orientation of encapsulated human bone marrow mesenchymal stem cells, whereas light-induced crosslinking maintained the aligned scaffold structure [23]. The regulation of network stress relaxation further promoted cell spreading, alignment, and ligament- or tendon-related differentiation [23]. However, the contribution of these cell-instructive properties to functional tissue regeneration remains to be validated in vivo. Multiple stimuli can couple local scaffold formation with externally or pathologically regulated delivery of regenerative agents. A thermoresponsive hydrogel underwent gelation close to body temperature after administration, while ultrasound provided an additional trigger for enhancing the release of bone morphogenetic protein 2 [142]. Ultrasound treatment increased growth-factor release from the pure hydrogel by up to 13.5-fold relative to its unstimulated control, while cumulative release reached 43.9% in the formulation containing the ultrasound-sensitive additive. Temperature responsiveness therefore supported local scaffold formation and retention, whereas ultrasound enabled externally controlled supplementation of the sustained growth-factor release. However, bone regeneration was not examined in a representative defect model [142]. In addition to externally triggered delivery, endogenous oxidative stress can similarly regulate therapeutic release during ischemic-tissue repair. A temperature- and ROS-responsive hydrogel underwent rapid gelation at 37 °C and increased ferulic-acid release under oxidative stress [144]. The released ferulic acid reduced ROS, inflammation, and apoptosis in endothelial cells, enabling enhanced angiogenic responses in a mouse hindlimb-ischemia model [144]. In this system, temperature responsiveness enabled rapid local gelation, whereas ROS responsiveness increased therapeutic release in the oxidative ischemic environment. Moreover, multi-responsive hydrogels can match the evolving requirements during tissue repair by assigning early microenvironment regulation and subsequent regenerative support to different responsive components and external triggers. For myocardial repair, a pH- and ROS-responsive hydrogel delivered curcumin-loaded NPs together with tailored recombinant humanized collagen type III [140]. The acidic and ROS-rich infarct environment accelerated hydrogel degradation and enabled relatively rapid curcumin release to reduce oxidative stress, inflammation, and apoptosis. The collagen component provided more sustained matrix and regenerative support for cell proliferation, migration, and angiogenesis. Through these complementary effects, the hydrogel improved cardiac function in a rat myocardial-infarction model [140]. A related strategy was applied to denervated muscle, where early oxidative and inflammatory damage and subsequent loss of cellular function require different forms of intervention. Bu et al. developed an ultrasound- and pH-responsive injectable hydrogel loaded with extracellular vesicles derived from human umbilical cord mesenchymal stem cells [139]. The carboxymethyl CS/chondroitin sulfate matrix initially provided anti-inflammatory and ROS-scavenging effects, while its pH-labile Schiff-base network supported injectable placement, local retention, and environment-dependent network remodeling. Ultrasound applied later during treatment disrupted reversible interactions within the hydrogel and increased extracellular-vesicle release. This externally regulated release maintained extracellular-vesicle bioactivity and improved the functional performance and morphological integrity of denervated muscle in a rat sciatic-nerve-injury model [139].

In addition to stage-dependent therapeutic delivery, multiple responsive mechanisms can also be coordinated with pre-engineered scaffold architectures to provide complementary structural and biochemical cues. Chen et al. constructed an extracellular matrix-mimicking neural scaffold containing a soft gelatin-based domain and a hard sulfated-cellulose-nanocrystal domain [60]. Directional freeze-casting and photocrosslinking produced aligned microchannels with a floor-pillar structure, which improved structural stability and provided directional guidance for cell organization, nutrient transport, and neurite extension. Sodium danshensu was immobilized through phenylboronate ester bonds and subsequently released in response to acidic pH, glucose, and $H_{2}O_{2}$. The released drug provided anti-inflammatory and antioxidant support, promoted oligodendrocyte maturation, and facilitated axonal extension and myelination together with the physical guidance supplied by the microchannels [60]. This system demonstrates how a preformed architecture can first provide directional support, while multi-responsive release supplies complementary biochemical signals. However, this coordination has so far been demonstrated mainly in vitro and requires validation in a nerve-injury model.

Collectively, multi-stimuli-responsiveness can contribute to tissue engineering through several complementary routes, including regulating cell-instructive structure and mechanics, and coordinating controlled delivery with changing therapeutic requirements. However, evidence for these functions in vivo remains uneven. Although functional improvement has been demonstrated in several ischemic, cardiac, and neuromuscular models [139,140,144], several systems have been evaluated mainly through material characterization, release studies, or short-term cell experiments [23,60,87,141,142]. Biosafety evaluation also varied substantially in scope, ranging from short-term in vitro cytocompatibility or haemocompatibility testing to local and systemic observations in small-animal models. Long-term local tissue responses, degradation-product safety, and systemic effects were not consistently evaluated. For tissue regeneration, the central challenge is not simply to preserve multiple responsive behaviours but to ensure that each response contributes a necessary function without compromising cell viability, nutrient transport, or scaffold integrity. To clarify how current systems address this challenge, Table 6 compares the tested stimulus ranges and key material and release response parameters of representative tissue-engineering hydrogels with their regenerative relevance, main regenerative outcomes, validation levels, and reported biosafety evidence. Future studies should establish the contribution of each response in representative defect models and evaluate long-term tissue integration and functional recovery.

## 4. Conclusions

Multi-stimuli-responsive hydrogels offer distinct biomedical advantages when different inputs are assigned to functionally necessary and experimentally distinguishable roles. Depending on the application, one input may regulate administration or local retention, whereas others may regulate therapeutic release through network remodeling, thereby enabling early microenvironment regulation, subsequent regenerative support, or externally controlled activation. Such benefits are conditional, however, and should not be inferred solely from the coexistence of multiple responsive components or therapeutic functions. Activation thresholds may shift after the responsive components are integrated into complete networks and exposed to protein- and ion-rich biological environments. The interaction among different components and constraints imposed by the network may also limit the translation of molecular changes into macroscopic hydrogel responses, thereby attenuating the observable network-level changes. The performance of a multi-responsive hydrogel should therefore be judged not only by the number or magnitude of its responses, but also by whether their coordination provides a demonstrable advantage over a single-responsive system.

A central limitation of current research is the gap between material responsiveness and biological requirements. The available evidence remains dominated by responses measured under simplified and fixed laboratory conditions that do not fully reproduce the heterogeneity and temporal evolution of relevant in vivo microenvironments. Many reported systems verify hydrogel sensitivity to each intended stimulus in separate material-level experiments, and some additionally examine the overall response behaviour under combined stimuli. However, these experiments usually confirm the presence of multiple responses without resolving the relationships among them. The available evidence is therefore often insufficient to distinguish independent, cooperative, and sequential effects or to quantify the contribution of each response to material and therapeutic performance. It may also remain unclear whether improved outcomes arise from multi-response coordination or primarily from the incorporated therapeutic agents. In addition, cascade or feedback mechanisms may sometimes be inferred from proposed reaction pathways or final treatment outcomes without direct experimental validation. Consequently, quantitative distinction among different response modes requires response-specific experimental designs. Cooperative responses should be assessed by comparing unstimulated, individual-stimulus, and combined-stimulus conditions to determine how the combined response differs from the individual responses. Sequential responses additionally require time-resolved measurements using different activation orders, together with controls involving simultaneous stimulation. For cascade and feedback mechanisms, disruption of intermediate steps and parallel monitoring of input signals and material outputs are required to verify causal transmission and feedback regulation. Depending on the proposed mechanism, changes in network mechanics can be characterized by oscillatory rheological measurements. Meanwhile, swelling kinetics can be tracked through time-dependent water uptake, whereas degradation kinetics can be evaluated through mass loss and changes in structural integrity. Time-resolved spectroscopy can follow stimulus-induced molecular transformations, whereas quantitative release measurements can assess the associated transport response. Correlation of these measurements and comparison of complete formulations with counterparts lacking selected responsive components can help determine the contribution of each response.

Beyond these experimental requirements, clinical translation of multi-stimuli-responsive hydrogels requires scalable and reproducible manufacturing, as well as regulatory evaluation. The compositional complexity of hydrogels may hinder scale-up and batch-to-batch reproducibility by introducing variations in component distribution, payload activity, and stimulus–response thresholds, thereby affecting overall performance. To reduce fabrication complexity and undesired interference among multiple components, future development should prioritize the minimum level of responsive complexity required for a defined biomedical objective. Quality control should verify conventional material properties, payload activity, and the preservation of the intended stimulus responses. For reversible systems, cycling tests should additionally determine whether response magnitude and network integrity are maintained during repeated activation. Performance should be examined within pathology-matched activation ranges and in representative biological media and disease models. Structural stability and component retention should also be assessed together with therapeutic performance. Further translational challenges arise from sterilization, regulatory evaluation, and long-term safety. The procedures reported in the reviewed studies mainly included UV irradiation, ethanol treatment, and filtration. However, these procedures were generally applied before cytocompatibility or antibacterial testing rather than being validated as terminal sterilization processes for the final products. This distinction is important because sterilization treatments involving radiation, oxidants, or organic solvents may alter or disrupt responsive structures, such as dynamic covalent junctions, thermoresponsive domains, and photosensitive groups. They may also damage sensitive biological payloads, including enzymes, exosomes, and growth factors. Future sterilization protocols should therefore be validated for both sterility assurance and preservation of the intended material and biological functions. Systems integrating drugs, biologics, or nanomaterials may further require product-specific regulatory evaluation based on defined composition, manufacturing controls, and the primary mode of action. Finally, cytocompatibility tests, body weight monitoring, and major-organ histology assessed over limited observation periods are insufficient to establish the long-term safety of functional nanocomposite hydrogels. Long-term safety assessments should examine the fate and toxicity of retained or released nanomaterials, metal ions, and degradation products, including their potential for organ accumulation and chronic local or systemic effects. Progress along these lines will be more important for the clinical translation of multi-stimuli-responsive hydrogels than the continued addition of responsive components without a clearly demonstrated functional necessity.

## Figures and Tables

**Table 1:** Representative molecular-level integration strategies for constructing multi-stimuli-responsive hydrogels.

Integrated Segments	Stimulus–Response Type	Relationship Between Responses	Biomedical Application	Ref.
Integration strategy: Copolymerization				
PNIPAM + Hy or maleic acid	Temperature + pH	Thermal gelation occurred without significant pH-dependent release at pH 6–8.	Local antimicrobial-peptide delivery for diabetic wounds	[41]
Lignin-p(NIPAm-*co*-AAc)	Temperature + pH	Alkaline pH promotes swelling, whereas thermally induced PNIPAM contraction suppresses it.	Potential biomedical scaffold	[89]
MeBSA + NIPAm	Temperature + pH	The pH-shifted PNIPAM thermal window is partially counteracted by MeBSA swelling.	Gastrointestinal drug delivery	[43]
mPEG-*b*-PMet	Temperature + ROS	PMet thermal association promotes gelation, whereas ROS oxidation enhances hydrophilicity and disrupts the association.	Local tumour chemoimmunotherapy	[21]
p(NIPAm-*co*-MAA-*co*-SPMA)	Temperature + pH + UV light	Low pH and temperature promote network expansion, with UV photoisomerization further enhancing drug diffusion.	DOX delivery	[76]
Integration strategy: Grafting				
Gelatin-*g*-OSM	Temperature + pH	Lower pH reduced the gelation temperature, whereas a higher OSM grafting level elevated the apparent pK_a_.	Local paclitaxel delivery following glioblastoma resection	[37]
CMC-*g*-(mPEG-*b*-PCL)	Temperature + pH	Lower pH, longer PCL blocks, and higher polymer concentration lowered the gelation temperature.	Injectable osteochondral scaffold	[26]
Tertiary-amine-modified mPEG–polypeptide	Temperature + pH	Higher pH and tertiary amines lowered the critical gelation concentration and temperature.	DOX delivery	[35]

Abbreviations: AAc, acrylic acid; DOX, doxorubicin; Hy, trans-*β*-hydromuconic acid; MAA, methacrylic acid; MeBSA, methacrylated bovine serum albumin; NIPAm, N-isopropylacrylamide; OSM, oligosulfamethazine; PCL, poly(*ε*-caprolactone); PMet, poly(L-methionine); SPMA, spiropyran methacrylate. In the polymer notation, *b*, *co*, *g*, and *p* indicate block linkage, copolymerization, graft linkage, and polymer, respectively.

**Table 2:** Quantitative response characteristics and experimental evidence for representative multi-stimuli-responsive hydrogels containing dynamic covalent bonds.

Dynamic Covalent Bond(s) [Ref.]	Bond-Associated Stimulus and Conditions	Other Responsive Mechanism and Conditions	Key Network Parameters	Release Parameters	Biological Outcome	Response-Mode Evidence and Level of Resolution
Boronate ester [22]	pH (5.0 vs. 7.2–7.4); Glucose (5 or 25 mM); H_2_O_2_ (1 or 5 mM)	—	Gelation: room temperature (RT); time not reported (NR). Swelling: NR. Disintegration increased at pH 5.0, with glucose (25 mM), and with H_2_O_2_ (5 mM).	Acidic pH and glucose enhanced TA release. H_2_O_2_-dependent release and quantitative cumulative values were NR.	Improved diabetic wound healing	NR: individual responses were tested separately and the inter-response relationship was unresolved.
Boronate ester [105]	pH (1, 3, 5, 7, or 11); Glucose (1.0, 1.8, 4.5, or 12 mg/mL)	—	Gelation: <10 s (RT). Swelling: NR. Gel strength: ∼0.65–32 kPa. Crossover modulus increased from ∼2.8 kPa (pH 1) to ∼5.4 kPa (pH 11).	At 300 min, release was 57%, 61%, 65%, and 98% with glucose at 1.0, 1.8, 4.5, and 12 mg/mL, respectively. Near-complete release occurred within 120 min in cell-culture medium.	Cytocompatibility and cell encapsulation demonstrated in vitro.	NR: individual responses were tested separately and the inter-response relationship was unresolved.
Semiclosed cyclic hemiaminal [106]	pH (≤6.5); GSH (100 mM); H_2_O_2_ (100 mM)	—	Gelation: 70 °C (1 h). Swelling: NR. $G^{\prime }$: ∼2740 Pa. Tensile stress: ∼300 kPa. Degradation: complete within 51 h (pH 6.5). Self-healing: 50 min at 37 °C.	Cumulative release reached 74.3% at pH 6.5. GSH- and H_2_O_2_-responsive bond degradation was demonstrated, but release under these stimuli was NR.	Accelerated infected and diabetic wound healing.	NR: individual responses were tested separately and the inter-response relationship was unresolved.
Diselenide [59]	DTT (10 mM) and H_2_O_2_ (0.5%)	Matrix–payload interactions: pH (5.0 vs. 7.4). Photosensitizer-mediated photothermal heating and ROS generation: NIR irradiation (808 nm, 4 W, 15 min)	Gelation: 155–509 s (RT). $G^{\prime }$: 75–300 Pa. Swelling (24 h): ∼900–1800% (PBS, RT). DTT (10 mM) caused ∼95% mass loss within 2 d.	Release reached 60% within 4 h at pH 5.0. Approximately 40%, 50%, and 60% released were reached after 8 h with DTT, H_2_O_2_, and NIR irradiation, respectively. Release approached 100% (120 h).	Enhanced in vitro tumour-cell inhibition	NR: individual responses were tested separately and the inter-response relationship was unresolved.
Phenylboronate ester [111]	pH (5.0 vs. 7.4) and H_2_O_2_ (1 mM)	Acid-induced micelle dissociation (pH 5.0 vs. 7.4)	Gelation: ∼3 s (RT). Swelling (12 h): ∼500% (pH 7.4, 37 °C). Degradation: <10% remaining after 3 days (pH 5.0 + H_2_O_2_). Self-healing: cut gels rejoin within 10 min.	Fast-release fraction (48 h): ∼50% (pH 5.0), 70% with 1 mM H_2_O_2_, and ∼90% under combined stimulation. Slow-release fraction: 60% at pH 5.0 and <30% with H_2_O_2_.	Improved infected diabetic wound healing.	Sequential: pH/ROS-triggered network disintegration followed by pH-triggered micelle release; experimentally tested using individual and combined stimuli and cargo-specific release profiles.
Disulfide [73]	DTT (concentration NR)	Light-responsive AB–CD association: UV light (365 nm) and visible light (450 nm)	Gelation: 90 °C for 10 min, then cooled to RT; gelation time NR. Swelling: NR. Mechanical: $G^{\prime }>G^{\prime \prime }$ at 0.5% strain (25 °C); after UV (365 nm).	UV or DTT increased cumulative release to ∼80% within 3–6 h and release remained <40% at 72 h without stimulation.	Maintained ∼98% cell viability.	NR: individual responses were tested separately and the inter-response relationship was unresolved.
Imine + Phenylboronate ester [103]	pH (5.4 vs. 7.4); H_2_O_2_ (0, 0.2, 1.0, or 2.5 mM)	—	Gelation: 9.6–14.3 s (RT). Swelling: NR. self-healing: rapid; cyclic recovery: 1–500% strain, 3 cycles, near-complete $G^{\prime }$ recovery. Acidic pH and H_2_O_2_ accelerated mass loss.	TOB release (160 h): 44.9% at pH 7.4; 60% at pH 5.4. EGCG release (130 h): 65.8% (0 mM H_2_O_2_), 67.5% (0.2 mM), 74.0% (1.0 mM), 81.3% (2.5 mM).	Improved diabetic wound healing with reduced scarring.	Selective: experimentally tested using cross-stimulus controls.
Imine + Disulfide [58]	Imine: pH (5.5, 6.5, and 7.4); Disulfide: GSH (1, 5 or 10 mM at pH 5.5)	Temperature-dependent hydrogen-bond disruption (25 vs. 37 °C)	Gelation: rapid at RT (time NR). pH-dependent swelling: ∼15–30% (40 h). Degradation increased at pH 5.5 and 37 °C. Mass loss was ∼11% under GSH (1 mM) at 37 °C.	At 48 h, release was 22.1%, 25.3%, and 28.5% at pH 7.4, 6.5, and 5.5, respectively. At pH 5.5, it reached 29.4%, 31.0%, and 32.7% with GSH at 1, 5, and 10 mM. Release was higher at 37 than 25 °C, but numerical values were NR.	Enhanced in vivo breast-tumour inhibition.	Cooperative pH and GSH response; combined pH and GSH conditions were experimentally tested, but their relationship with temperature was not quantitatively resolved.
Schiff base + Phenylboronate ester [12]	Schiff base: pH (6.5 vs. 7.4); Phenylboronate ester: pH (6.5 vs. 7.4) and glucose (1 and 3 mg/mL)	GOX-mediated acidification and CAT-mediated H_2_O_2_ decomposition: glucose (3 mg/mL); initial pH 7.4.	Gelation: 10–20 s at RT. Swelling: ∼3000% (12 h, pH 7.4, 37 °C). Degradation: ∼70% mass retained after 10 days. Lower pH and higher glucose caused visible disintegration (parameters NR).	At 36 h, release was ∼30% at pH 7.4, ∼45% and ∼56% with glucose at 1 and 3 mg/mL, respectively, and ∼69% at pH 6.5.	Improved diabetic wound healing.	Cascade: a glucose-initiated enzymatic cascade and feedback loop were proposed. Cascade outputs were experimentally measured, but individual bond-cleavage kinetics and reversed-order controls were NR.

Note: NR: not reported in the original study. RT: room temperature. Response-mode evidence records only relationships explicitly proposed in the original study. Level of resolution indicates whether the inter-response relationship was experimentally tested or remained unresolved.

**Table 3:** Nanomaterial-specific activation, transduction, and biological evidence for representative multi-stimuli-responsive hydrogels.

Nanomaterials and Loading Fraction	Function	Activated Conditions	Transduction Parameters	Biocompatibility, Retention, and Leaching Evidence	Ref.
CuS (100μg/mL) + AuNPs (0.05mg/mL)	Photothermal + Structural crosslinker	NIR (650–1000 nm, 533 mW, 15 min); DTT (0.5 M, 37°C); GSH (0.1 M, 37°C)	Photothermal enhancement: ΔT=19.5°C (15 min); NIR-triggered gelation occurred within 15 min.	CuS@SPPR showed enhanced cytotoxicity toward breast cancer cells. Only cancer cells were tested. Normal cells and complete hydrogels were NR.	[72]
AuNS@PVP + AgNCs [0.02 or 0.08% (*w*/*v*) ]	Photothermal + Structural modifier	Heating (37°C, 3 min); light irradiation (AuNS@PVP, 200–700 nm; AgNCs, 400–500 nm; 90 s or 10 min)	After 10 min of irradiation at 37°C, the as-prepared/swollen hydrogels reached 39.2/39.7 °C for AuNS@PVP and 39.4/42.2 °C for AgNCs.	Cell viability: >82% (24 and 48 h), reaching >100% for selected NP-containing formulations (24 h). No NP release was detected (3–72 h), and no metal-ion release or inhibition zones were observed (24 h).	[14]
PDA NPs (0.6 wt%)	Photothermal + Structural Filler	NIR (808 nm, $4\;W/{\mathrm{cm}}^{2}$, 5 min)	Temperature reached 63°C after 5 min in vitro and 67.3°C in vivo. Repeated irradiation caused wound scorching.	Cell viability: ∼96% for the hydrogel extract (48 h). In vivo: stable body weight, reduced blood-cell counts and wound scorching after repeated irradiation (21 days).	[114]
CaCO_3_/PDA NPs (500μg/mL)	Photothermal + Structural crosslinker + Ion-releasing unit	NIR (808 nm, $1.0\;W/{\mathrm{cm}}^{2}$; 10 min for photothermal conversion and Ca^2+^ release; 8 min for cell tests); pH 6.5	Temperature increase: 28.1 °C (10 min); photothermal conversion efficiency: 39.25%; stable photothermal response over three on/off cycles. Tumour-site temperature: ∼56°C (in vivo, 8 min).	Cell viability: no significant reduction (48 h); negligible haemolysis. In vivo: stable body weight, normal blood counts, and no major-organ damage (14 days). Greater tumour retention and lower major-organ distribution of hydrogel-embedded NPs than free NPs (72 h).	[115]
Fe_3_O_4_ NPs (0.1 wt%)	Photothermal + Nanozyme + Structural filler	NIR (808 nm, $1\;W/{\mathrm{cm}}^{2}$, 5 min); Fenton reaction (pH 6.5, 100 mM H_2_O_2_; 37 or 45°C)	Photothermal enhancement: ΔT=9.0°C (5 min); tumour-site temperature: 41.7 °C (in vivo, 5 min). Fenton-mediated ·OH generation increased at 45 °C.	Cell viability: >80% (24 and 48 h); haemolysis: <5% (2 h). In vivo: stable body weight and no major-organ lesions (13 days).	[56]
COFZn@TA NPs (400μg/mL)	Photothermal + Nanozyme + Structural crosslinker	NIR (808 nm, $1\;W/{\mathrm{cm}}^{2}$, 5 min); glucose-containing serum (37°C, 24 h); intracellular ROS/redox conditions	Wound-site photothermal enhancement: ΔT=31.7–33.4°C, reaching 59.0–59.4 °C (in vivo, 5 min). GOX-like activity decreased serum glucose in a concentration-dependent manner (24 h).	Cell viability: ∼90% (test duration NR); no noticeable haemolysis (4 h). Major-organ histology: no lesions (3 days).	[24]
Au/TF-MOF nanosheets [2mg/mL (0.2 wt%)]	Nanozyme + Sonosensitizer	Ultrasound (1 MHz, $2\;W/{\mathrm{cm}}^{2}$, 5 min); H_2_O_2_ (10 mM for CAT-like activity and 100 mM for peroxidase-like activity, 37°C); pH 5.0–7.4	Ultrasound-induced O21 generation increased with nanosheet concentration (0.5–2mg/mL) and power density (1–$2.4\;W/{\mathrm{cm}}^{2}$).	Cell viability: >80% (24 and 48 h); haemolysis: <5% (2 h). In vivo: stable body weight and normal major-organ histology (13 days).	[17]
bTiO_2_ NPs (1 wt%)	Sonosensitizer + Photosensitizer + Structural crosslinker	Ultrasound (1 MHz, $1\;W/{\mathrm{cm}}^{2}$, 50% duty cycle, 5 min); NIR (808 nm, $1\;W/{\mathrm{cm}}^{2}$, 5 min); pH 5.5–7.4	Photothermal enhancement: ΔT=15°C in PBS (5 min); temperature reached ∼45 °C in bacterial suspension (5 min). Combined stimulation enhanced O21 and ·OH generation and 48% of the ·OH signal was retained.	Cell metabolic activity: ∼85% of the control (day 1), with continued proliferation through day 5. Combined ultrasound and NIR treatment reduced metabolic activity to ∼65% of the control (24 h).	[15]
TiO_2_@MS-SH NPs (3 wt%)	Sonosensitizer + Structural crosslinker	Ultrasound (120 kHz; power NR; 10 min for secondary crosslinking and 10–60 min for antibacterial treatment); pH 5.5–7.4	Ultrasound generated O21 and triggered thiol–norbornene crosslinking. O21 production increased with sonication time and was highest at 3 wt% loading.	Cell metabolic activity: ∼3.0-fold increase (days 1–7); predominantly viable cells (day 7; in vitro only).	[118]
Fe_3_O_4_ NPs (3, 6, or 9 wt%)	Magnetic Response Unit + Structural Filler	Static magnetic field (strength and exposure duration were NR). pH 5.0, 6.5, or 7.4 (37°C);	Saturation magnetization increased from 1.38 to 2.25emu/g as Fe_3_O_4_ loading increased from 3 to 9 wt%.	No biological testing was performed.	[121]
MNPs or MNP@DMA [0.5% (*w*/*v*)]	Magnetic alignment unit + Structural crosslinker	Static magnetic field (35±2 mT, 3 min); UV (365 nm, $20\;\mathrm{mW}/{\mathrm{cm}}^{2}$, 3 min)	NP alignment began (1–5 s) and became clearly evident (10 s). Simultaneous UV curing fixed the anisotropic structure, which remained stable (28 days).	High cell viability (day 28); metabolic activity increased 4–7-fold (days 0–28). NP chains remained aligned within the hydrogels (day 28; in vitro only).	[23]
MoS_2_/Fe_3_O_4_ nanocomposite (43±2% Fe_3_O_4_ surface coverage; hydrogel loading NR)	Magnetic alignment Unit + Photothermal + Structural unit	Magnetic field (1 T, 10 s for voxel orientation or 24 h for bulk orientation); NIR (808 nm, $2.5\;W/{\mathrm{cm}}^{2}$, up to 1200 s)	Saturation magnetization: 47.6emu/g. Photothermal enhancement (2 min): ΔT∼35°C (perpendicular orientation) and ∼24°C (parallel orientation), reaching 55.2 and 44.1°C, respectively.	No biological testing was performed.	[113]

General evidence limitation: Only evidence explicitly reported in the primary studies is included. Unless otherwise stated, quantitative data on long-term nanomaterial retention or unintended leaching, intact-particle clearance pathways, and long-term biosafety were not reported.

**Table 4:** Quantitative response characteristics, pathological relevance, and therapeutic and safety evidence for representative multi-stimuli-responsive hydrogels in wound treatment.

Stimuli and Tested Ranges	Key Network Parameters	Release Parameters	Pathological Relevance	Main Therapeutic Outcome	Validation Level	Reported Biosafety Evidence	Ref.
pH (5.0 vs. 7.4) + glucose (10 or 40 mM)	Gelation: 40–75 s at RT. Swelling (6 h): ∼107–115%. Hydrogel remaining (day 14): 70% (10 mM glucose), 62% (40 mM glucose), and 40% (pH 5.0). Cyclic recovery: remained $G^{\prime }$ > $G^{\prime \prime }$ after five recovery cycles.	Pravastatin/peptide release (day 5): 50.2%/64.4% (pH 7.4), 64.5%/73.6% (40 mM glucose), and 80.8%/83.7% (pH 5.0).	pH 5.0: may mismatch the alkaline conditions of chronic diabetic wounds. Glucose: 10–40 mM spans hyperglycaemic to high-challenge conditions.	Wound closure: 70% (day 7) and nearly complete (day 14). Wound bacterial survival: <5%.	Infected diabetic-rat foot wound	Cell proliferation: ∼120% (72 h). Haemolysis: <5%.	[127]
Temperature (25–42 °C) + NIR light (808 nm, $2\;W/{\mathrm{cm}}^{2}$, 10 min)	Gelation: RT (time NR). Swelling (72 h): ∼70–85% (PBS, 37 °C). Compressive strength: 0.18 MPa. Temperature response: resistance change up to 35% over 25–42 °C, maintained over 10 cycles.	NO release: NIR-controlled on–off release (>300 μM).	Temperature: tracks wound inflammation. NIR-triggered NO: provides antibacterial and anti-inflammatory effects.	Wound closure: nearly complete (day 10). Tissue response: reduced inflammation and enhanced repair.	Full-thickness skin wound in healthy rats	Cell proliferation: maintained through day 7.	[123]
NIR light (808 nm, $0.25\;W/{\mathrm{cm}}^{2}$) + alternating magnetic field (AMF, 1 kW, 120 s)	Gelation: UV irradiation (1 min) and Ca^2+^ crosslinking (0.2% for 5 min). Tensile strength: ∼4.1 kPa at 55% strain. Thermal response: 41 °C (NIR, 60 s) and 39 °C (AMF, 120 s), with responses maintained over five cycles.	AgNP release: ∼60% after five NIR cycles.	Remote heating: triggers antibacterial AgNP release for infected superficial and deep wounds.	Wound closure: 97% by day 12. Deep infection: reduced inflammation and infection by day 8.	Infected diabetic-rat skin and subcutaneous wounds (NIR: in vivo; AMF: in vitro only)	Cell viability: >95% (day 3).	[18]
pH (5.0 vs. 7.4) + ROS (H_2_O_2_, 1 mM)	Gelation: RT (time NR). Swelling ratio (12 h): 3.0–3.3 (pH 7.4, 37 °C). Degradation: ∼70% by day 7 (pH 7.4, 37 °C) and disintegration within 6 h (pH 5.0 or 1 mM H_2_O_2_). Self-healing: rapid recovery during repeated 1–1000% step-strain switching; cut gels rejoined within 2 min.	DS release (24 h): 58.6% (pH 5.0), 63.8% (1 mM H_2_O_2_), and 84.5% (combined stimuli). MF release (24 h): 22.6% (pH 7.4) and 36.9% (pH 5.0), followed by sustained release over 7 days.	pH 5.0: may mismatch the alkaline conditions of chronic diabetic wounds. ROS: relevant to oxidative stress.	Wound closure: 76.5% (day 7) and nearly complete (day 14). Bacterial inhibition: 91.1–97.3%.	Infected diabetic-rat full-thickness skin wound	Cell viability: >90% (day 3). Haemolysis: ≤3%.	[128]
pH (5.0/5.5 vs. 7.4) + ROS (H_2_O_2_, 0.1 or 1 mM)	Gelation: RT (time NR). Swelling (12 h): ∼250%. Degradation: disintegration within 6 h at pH 5.0 or 1 mM H_2_O_2_. Cyclic recovery: rapid recovery during two 1–1000% step-strain cycles; cut gels self-healed within 2 min. Compression: ∼22 kPa at 80% strain without fracture.	Astragaloside release (24 h): 26.3% (pH 7.4), 37.9% (pH 5.5), 50.4% (0.1 mM H_2_O_2_), and 59.1% (combined stimuli), followed by sustained release over 72 h.	pH 5.0–5.5: may mismatch the alkaline conditions of chronic diabetic wounds. ROS: relevant to oxidative stress.	Wound closure: 94.7% (day 21). Bacterial inhibition: 91.6–95.3%.	Infected-rat full-thickness skin wound	Cell viability: >90% (day 3). Haemolysis: no significant increase.	[125]
pH (5.5 or 6.5 vs. 7.4) + lipase/lactic acid (6 mM)	Gelation: NR. Degradation: >250 h. Self-healing: rapid recovery under alternating shear strain; cut gels rejoined within 30 min (37 °C).	Catalytic-component release (168 h): 53.2% (pH 7.4) to 98.1% (pH 5.5). Cascade products (pH 5.5): 1.26 mM H_2_O_2_ (20 min) and 8.2μM NO.	Acidic pH and lipase: reflect bacterial metabolism, although pH 5.5 may mismatch the alkaline conditions of chronic diabetic wounds.	Wound closure: 64.3% (day 5), 93.8% (day 10), and nearly complete (day 15). Bacterial inhibition: 96.4–99.4%.	Infected mouse-neck full-thickness wound	Cell viability: >90%. Haemolysis: <5%.	[124]
pH (7.4–9.0) + ROS (H_2_O_2_, 5 or 10 mM in vitro)	Gelation: overnight (37 °C), with complete crosslinking within 2–3 h at pH 7.4. Adhesion retention (48 h): 92.1±3.2% on wounds. Degradation: 30% in PBS and 36% in simulated wound fluid over 4 weeks. Cyclic recovery: complete recovery after 1000% strain for at least four cycles.	Antimicrobial/nanozyme release (24 h): 45.6%/42.1% (pH 7.4), 62.5%/58.3% (pH 8.5), and 72.3%/68.9% (10 mM H_2_O_2_, pH 7.4). H_2_O_2_ decomposition: 98.7% at 24 h. Catalytic durability: 88.5±0.3% after 10 cycles in simulated wound exudate.	Alkaline pH: overlaps the reported diabetic-wound range (pH 7.2–8.5). H_2_O_2_: represents oxidative stress and enables oxygen generation.	Wound area remaining: 20.89% (day 8) vs. 69.9% (control). Bacterial inhibition: >99.9%.	Infected diabetic-mouse full-thickness wound	Cell viability: ∼100% (24 h). Haemolysis: <5%. In vivo safety: stable body weight and normal major-organ histology (day 28).	[129]

**Table 5:** Quantitative response and release characteristics, physiological or pathological relevance, and therapeutic and safety evidence for representative multi-stimuli-responsive hydrogels used for therapeutic delivery.

Stimuli and Tested Ranges	Key Network Parameters	Release Parameters	Pathological Relevance	Main Therapeutic Outcome	Validation Level	Reported Biosafety Evidence	Ref.
pH (1.2 vs. 6.8) + redox (125 mM DTT)	Gelation: overnight (RT). pH/redox-dependent swelling: q≈2.2–16.0 (6 h, 37 °C). Network collapsed at pH 1.2 and swollen at pH 6.8. Complete disintegration with DTT (12 h).	Mg^2+^release: 20.4% (pH 1.2), 30.8% (pH 6.8), and 72.5% (pH 6.8 + DTT, 6 h).	pH: reflects gastric-to-intestinal passage. Redox: 125 mM DTT is a nonphysiological reducing surrogate.	Site-selective intestinal Mg^2+^ delivery.	In vitro only: simulated gastrointestinal fluids	Cell viability: >95% (24 h).	[10]
pH (1.2, 6.5, or 7.4) + bacterial enzymes (2% rat cecal content at pH 6.5)	Swelling: increased with pH. Gelation and degradation: quantitative parameters were NR.	Metronidazole release: minimal at pH 1.2, ∼80% at pH 6.5, and 100% at pH 7.4. Rat cecal content accelerated release at pH 6.5.	The pH sequence and bacterial-enzyme trigger are relevant to colonic delivery.	Restricted upper-gastrointestinal release and accelerated colonic release.	In vitro release + ex vivo rat cecal-content assay	Biosafety data were NR.	[9]
Temperature + pH (7.4, 6.6, or 5.2) + ROS (H_2_O_2_, 400μM)	Gelation: 32.7±2.4°C (time NR). Swelling: ∼34% (pH 7.4, 37 °C).	Quercetin/Zn^2+^ release (96 h): 50%/26μg/mL (pH 7.4), 60%/43μg/mL (pH 6.6), 65%/48μg/mL (pH 6.6 + H_2_O_2_), and 80%/60μg/mL (pH 5.2).	pH 6.6: matches inflamed synovial fluid (pH 6.6–7.2). Gelation below 37 °C enables injection followed by intra-articular retention. H_2_O_2_: 400μM models cellular oxidative stress.	Reduced synovial inflammation and cartilage degradation; promoted cartilage repair.	Intra-articular treatment: rat osteoarthritis model	Cell viability: >90% (48 h). Major-organ histology: no lesions (day 28).	[36]
pH (4.0, 7.5, or 10.0) + glucose (100 or 500 mM)	Gelation: within 6 h at RT. Swelling: NR. Network response: $G^{\prime }$ decreased to 0.81-fold (pH 4.0) and 0.20-fold (100 mM glucose), with complete dissolution within 24 h in 100 or 500 mM glucose. Mechanical recovery: recovery during alternating 0.5–50% step-strain; cut gels healed within 4 h.	Insulin release (24 h, 100 mM glucose): 97%/51%/48% at pH 4.0/7.5/10.0 (glucose-dependent increase: 50%/26%/27%).	Glucose: 100–500 mM represents supraphysiological challenge conditions. pH: only pH 7.5 matches the subcutaneous environment.	Blood glucose decreased from 400 to 122 mg/dL within 4 h and remained lower than that with free insulin for 24 h.	In vitro and in vivo: subcutaneous administration in type-1 diabetic rats	No significant cytotoxicity; major-organ histology showed no tissue injury.	[8]
Glucose (1 or 4 g/L) + pH (7.4 vs. 4.5)	Gelation: 2–5 min at RT. Network response: contraction at pH 4.5 and pore enlargement at pH 7.4 in 4 g/L glucose.	Insulin release (12 h, pH 7.4): 6.0/6.2/8.7μg at 0/1/4 g/L glucose. Reversible pulsatile release was maintained over three alternating glucose and pH cycles.	Glucose: 1–4 g/L spans normoglycaemic to severe hyperglycaemic conditions. pH: 7.4 matches the subcutaneous environment, whereas pH 4.5 does not.	Glucose- and pH-regulated pulsatile insulin release.	In vitro response and release test only	Cell viability: >80% (24 h). Free responsive polymers caused 72.1–85.1% haemolysis at pH 6.0.	[51]
Temperature (25 vs. 37 °C) + pH (7.4 vs. 6.5)	Sol–gel transition: 27 °C (pH 7.4) and 25 °C (pH 6.5). Swelling: NR. Hydrogel remaining after 7 days in vivo: 54%, with complete degradation within 3 weeks.	Paclitaxel release (day 9): 71% (pH 7.4) and 63% (pH 6.5).	Temperature: transition below 37 °C supports in situ gelation. pH 6.5: matches the acidic tumour environment.	Suppressed orthotopic glioblastoma growth.	In vitro cell testing and in vivo: orthotopic glioblastoma xenograft mouse model	Cell viability: >96% (48 h). No visible inflammation was observed over 3–4 weeks.	[37]
Temperature (20–26 °C) + ROS (1 or 10 mM H_2_O_2_)	Gelation: 20–26 °C. Swelling: NR. Degradation: complete within 16/5 days in 1/10 mM H_2_O_2_.	Drug release (5 days): >80% in 10 mM H_2_O_2_. Antibody release: >85% within 13/5 days in 1/10 mM H_2_O_2_.	Temperature: transition below 37 °C supports in situ gelation. ROS: 1–10 mM H_2_O_2_ represents supraphysiological challenge conditions.	Suppressed tumour growth and postoperative recurrence, prolonged survival, and induced antitumour immune memory.	In vitro cell testing and in vivo melanoma-bearing and postoperative-resection mouse models	Cell viability: >80% (24 h). In vivo: stable body weight, normal major-organ histology, and no adverse local tissue reactions during 10-week degradation.	[21]
pH (6.5 vs. 7.4) + intracellular redox (GSH)	Gelation: <0.5 min with 10 mM CaCl_2_ (temperature NR). Swelling: NR. Young’s modulus: ∼0.55 MPa. Degradation: accelerated at pH 6.5 and nearly complete in vivo (day 10).	Prodrug/antibody release (day 10, pH 6.5): ∼65%/86%.	pH 6.5: matches the acidic postoperative tumour environment. GSH: intracellular redox tumour-associated activation.	Suppressed postoperative recurrence and distal tumour growth and prolonged survival.	In vitro cell testing and in vivo post-resection melanoma and bilateral-tumour mouse models	Cell viability: >90% (24 h). In vivo: stable body weight and normal major-organ histology over 21 days.	[135]
pH (5.0, 6.8, or 7.4) + H_2_O_2_ (100μM) + GSH (100μM or 10 mM) + glucose (1 mg/mL)	Gelation: Gelation: ∼45–50 min (37 °C, pH 7.4). $G^{\prime }$: 1.0–1.1 kPa. Swelling: NR. Degradation: complete within 7 days at pH 7.4 and within 3–4 days at pH 6.8 with 100μM H_2_O_2_ or GSH.	Tirapazamine/ GOX release (day 3): ∼56%/46% without glucose, >80% with glucose, and 95%/86% with 10 mM GSH.	pH 6.8 and glucose: match the tumour extracellular environment. H_2_O_2_ and GSH: model oxidative stress and intracellular reduction, respectively.	Enhanced tumour starvation and hypoxia-activated chemotherapy, suppressing tumour growth and prolonging survival.	In vitro cell testing and in vivo subcutaneous melanoma mouse model	Cell viability: >90% (24 h). In vivo: body weight increased and no evident major-organ damage was observed over 9 days.	[64]
Temperature (25–100 °C) + NIR light (1064 nm)	Sol–gel transition: ∼45 °C (time NR). Swelling: NR. NIR heating: 25 to 78 °C in vitro ($1.2\;W/{\mathrm{cm}}^{2}$, 5 min); tumour temperature reached 45 °C ($0.75\;W/{\mathrm{cm}}^{2}$, 100 s).	Quercetin release: ∼8% during the first 5 min of irradiation and 32% after four on–off cycles.	NIR irradiation: $0.75\;W/{\mathrm{cm}}^{2}$ for 5 min produced a tumour temperature of ∼45 °C, consistent with mild photothermal treatment.	Suppressed heat-shock response and enhanced photothermal tumour ablation.	In vitro cell testing and in vivo subcutaneous breast-tumour mouse model	Cell viability: >90% (24 h). In vivo: stable body weight and normal major-organ histology over 14 days.	[138]

**Table 6:** Quantitative material and release responses, regenerative relevance, and experimental and safety evidence for representative multi-stimuli-responsive hydrogels in tissue engineering.

Stimuli and Tested Ranges	Key Network and Release Parameters	Regenerative Relevance	Main Regenerative Outcome	Validation Level	Reported Biosafety Evidence	Ref.
Temperature (>95 to 25 °C) + pH (9.0 to 7.4) + ions (0.01 M PBS)	Gelation: 30 min after sequential temperature, ion, and pH switching. Swelling: NR. Storage modulus: up to ∼8.2 kPa (pH 7.0, 37 °C). Self-recovery: reversible gel–sol transition between 0.5% and 100% strain. Degradation: moderate mass loss in PBS at 37 °C.	The final pH and temperature are physiologically compatible, although the initial heating and alkaline dissolution are ex vivo fabrication conditions.	Promoted chondrocyte proliferation, matrix secretion, and phenotype maintenance.	In vitro: cartilage-cell scaffold. Non-representative in vivo evidence: rabbit defects used only to assess injectability.	Cell viability: >80% (72 h); cell attachment and proliferation were maintained through day 7.	[141]
Magnetic field (35±2 mT) + UV light (365 nm, $20\;\mathrm{mW}/{\mathrm{cm}}^{2}$, 3 min)	Magnetic alignment: 20–60 s. Photogelation: 1–3 min under UV irradiation. Swelling: ∼33–37% (1 day) and ∼52–65% (28 days) (PBS, 37 °C). Storage modulus: 1.07–1.13 kPa. Self-recovery/healing: rapid recovery under alternating 1–500% strain and reconnection (3 h).	The mild static field and short UV exposure enabled cell-compatible scaffold formation.	Directed cell alignment and promoted tendon- or ligament-related differentiation.	In vitro only: cell-laden three-dimensional constructs	High cell viability was maintained, with metabolic activity increasing 4–7-fold by day 28.	[23]
Temperature (20–50 °C) + ultrasound ($9.6\;\mathrm{mW}/{\mathrm{cm}}^{2}$, 2.5 min/h for 5 h)	Gelation: <37 °C (time NR). Swelling: NR. Injectability: <6.5 N. Repeated activation: five hourly ultrasound pulses; effective triggering was retained after 72 h. Growth-factor release (77 h): 43.9% with vs. 26.7% without the ultrasound-sensitive additive.	Body-temperature gelation supports local retention, and ultrasound enables timed release.	Provided sustained and ultrasound-enhanced growth-factor release.	In vitro only: release in physiological buffer and no bone-defect validation	NR.	[142]
Temperature (25–37 °C) + ROS (600μM H_2_O_2_)	Gelation: at 37 °C (10 s). Swelling: NR. Degradation: >14 days in vivo. Storage modulus: ∼0.4 MPa (37 °C). ROS scavenging: 66.9% (600μM H_2_O_2_). Therapeutic-agent release: >15% with H_2_O_2_ at 24 h vs. <15% without H_2_O_2_ by day 7.	Body-temperature gelation supports injectable retention, and 600μM H_2_O_2_ represents a relatively high in vitro oxidative challenge.	Reduced oxidative injury and enhanced angiogenesis in ischemic tissue.	In vitro oxidative-stress cell testing. In vivo rabbit subcutaneous implantation and mouse hindlimb-ischaemia model	Cell viability: >90% (48 h). No haemolysis, inflammation, or fibrosis was observed through day 14.	[144]
pH (5.0 vs. 7.4) + H_2_O_2_ (0.2 mM)	Gelation: 30 °C (time NR). Swelling: NR. Storage modulus: ∼70 Pa (37 °C). Self-recovery: rapid recovery during alternating 1–1000% strain. Degradation: ∼90% under combined stimuli by day 3 and nearly complete at pH 7.4 by day 7. Drug/collagen release (day 1, pH 7.4): 16.5%/10.1%, with the fastest release under combined stimuli.	Acidic pH and ROS model the infarct environment, but the tested levels were not verified in infarct tissue.	Reduced early tissue injury and improved cardiac function and angiogenesis.	In vitro cell testing and in vivo rat myocardial-infarction model evaluated for 14 and 28 days	Cell viability was maintained through 72 h. Haemolysis: ∼0.5%.	[140]
pH (6.0 vs. 7.4) + ultrasound (1 MHz, 45 W, 10 min in vitro; 15 min in vivo)	Gelation: conditions NR. Swelling (8 h): ∼5000%. Heating: 25 to 39 °C after 10 min. Storage modulus: ∼200 Pa before and 150 Pa after ultrasound. Swelling ratio (10 h): ∼52 without and 65 after ultrasound. Enzymatic degradation (day 6): ∼57% without and 69.9% after ultrasound. Repeated activation/release (420 min): reaching 92.4% under combined stimuli after four repeated 10 min ultrasound treatments.	pH 6.0 models early local acidity but was not verified in denervated muscle, while ultrasound enables externally timed release.	Preserved muscle morphology and restored muscle strength, circumference, and wet weight.	In vitro cell testing and in vivo rat sciatic-nerve injury with denervated-muscle evaluation for up to 12 weeks	Cell response: attachment and proliferation were maintained during 3-day culture. Degradation: 69.9% in lysozyme-containing buffer by day 6.	[139]
Glucose (5.56 or 22.20 mM) + pH (5.0–7.4) + H_2_O_2_ (1 or 4 mM)	Photocrosslinking: 405 nm, 20 °C, 30 min. Swelling (24 h): 1500–3500%. Degradation (day 18): 25–30%. Therapeutic-agent release (216 h): 36.5% (pH 7.4), 63.5% (22.20 mM glucose), 63.3% (pH 5.0), and 73.3% (4 mM H_2_O_2_).	Acidic pH and ROS model inflammatory neural injury, but glucose relevance was not established. H_2_O_2_ at 1–4 mM represents a high in vitro challenge.	Promoted cell orientation, oligodendrocyte maturation, axonal extension, and myelination.	In vitro only: cell compatibility through 7 days and neuron–glial coculture/myelination for 9 days. No nerve-injury validation	Cell viability remained above the control level through day 7. Degradation: 25–30% mass loss in PBS by day 18.	[60]

## Data Availability

No new data were created or analyzed in this study. Data sharing is not applicable to this article.

## Abbreviations

The following abbreviations are used in the manuscript. Chemical formulae, elemental symbols, ionic species, mathematical symbols, and units are not included. Material labels appearing only in cited figures retain their original source notation.

**Abbreviation**	**Full term**
AAc	Acrylic acid
AB	Azobenzene
AgNCs	Silver nanocubes
AgNP(s)	Silver nanoparticle(s)
AMF	Alternating magnetic field
AuNPs	Gold nanoparticles
AuNS	Gold nanospheres
Au/TF-MOF	Gold-decorated titanium/iron metal–organic framework
bTiO_2_	black TiO_2_
CAT	Catalase
CD	Cyclodextrin
CMC	Carboxymethyl cellulose
COF(s)	Covalent organic framework(s)
COFZn	Zinc-coordinated porphyrin covalent organic framework
COPMA	Methacrylated collagen peptide
CS	Chitosan
DMA	Dopamine methacrylamide
DNA	Deoxyribonucleic acid
DOX	Doxorubicin
DS	Diclofenac sodium
DTT	Dithiothreitol
EGCG	Epigallocatechin-3-gallate
ELP(s)	Elastin-like polypeptide(s)
GOX	Glucose oxidase
GSH	Glutathione
HA	Hyaluronic acid
HA-His	Histamine-modified hyaluronic acid
IPN(s)	Interpenetrating polymer network(s)
LCST	Lower critical solution temperature
MAA	Methacrylic acid
MeBSA	Methacrylated bovine serum albumin
MF	Mangiferin
MMPs	Matrix metalloproteinase(s)
MNP(s)	Magnetic nanoparticle(s)
MOF	Metal–organic framework
MS-SH	Thiolated mesoporous silica
mPEG	Methoxy poly(ethylene glycol)
NIPAm	N-Isopropylacrylamide
NIR	Near-infrared
NO	Nitric oxide
NP(s)	Nanoparticle(s)
NR	Not reported
OAL-CS	Allyl chitosan
OSM	Oligosulfamethazine
PAA	Poly(acrylic acid)
PBA	Phenylboronic acid
PBS	Phosphate-buffered saline
PCL	Poly(ε-caprolactone)
PDA	Polydopamine
PDMAEMA	Poly[2-(dimethylamino)ethyl methacrylate]
PEG	Poly(ethylene glycol)
PLL	Poly(L-lysine)
PLP	Poly(L-lysine isophthalamide)
PMAA	Poly(methacrylic acid)
PMet	Poly(L-methionine)
PNIPAM	Poly(N-isopropylacrylamide)
PNIPAM-SH	Dithiol-terminated poly(N-isopropylacrylamide)
PSA	Poly(sodium acrylate)
PVA	Poly(vinyl alcohol)
PVP	Poly(vinylpyrrolidone)
ROS	Reactive oxygen species
RT	Room temperature
semi-IPN(s)	Semi-interpenetrating polymer network(s)
SG	Succinoglycan
SP	Spiropyran
SPMA	Spiropyran methacrylate
SPPR(s)	Self-assembled thiol-rich polypseudorotaxane(s)
TA	Tannic acid
TF	Titanium/iron
TK	Thioketal
TOB	Tobramycin
UCST	Upper critical solution temperature
UV	Ultraviolet
